# Cancer-associated fibroblasts induce metalloprotease-independent cancer cell invasion of the basement membrane

**DOI:** 10.1038/s41467-017-00985-8

**Published:** 2017-10-13

**Authors:** Alexandros Glentis, Philipp Oertle, Pascale Mariani, Aleksandra Chikina, Fatima El Marjou, Youmna Attieh, Francois Zaccarini, Marick Lae, Damarys Loew, Florent Dingli, Philemon Sirven, Marie Schoumacher, Basile G. Gurchenkov, Marija Plodinec, Danijela Matic Vignjevic

**Affiliations:** 1 Institut Curie, PSL Research University, CNRS, UMR 144, F-75005 Paris, France; 2 Biozentrum, University of Basel, CH-4056 Basel, Switzerland; 3 Institut Curie, Department of surgical oncology, Curie Institute, F-75005 Paris, France; 4 Institut Curie, PSL Research University, Laboratoire de spectrométrie de masse protéomique, F-75005 Paris, France; 5 Institut Curie, PSL Research University, CNRS, U830, F-75005 Paris, France; 6 Institute of Pathology, University Hospital Basel, CH-4031 Basel, Switzerland

## Abstract

At the stage of carcinoma in situ, the basement membrane (BM) segregates tumor cells from the stroma. This barrier must be breached to allow dissemination of the tumor cells to adjacent tissues. Cancer cells can perforate the BM using proteolysis; however, whether stromal cells play a role in this process remains unknown. Here we show that an abundant stromal cell population, cancer-associated fibroblasts (CAFs), promote cancer cell invasion through the BM. CAFs facilitate the breaching of the BM in a matrix metalloproteinase-independent manner. Instead, CAFs pull, stretch, and soften the BM leading to the formation of gaps through which cancer cells can migrate. By exerting contractile forces, CAFs alter the organization and the physical properties of the BM, making it permissive for cancer cell invasion. Blocking the ability of stromal cells to exert mechanical forces on the BM could therefore represent a new therapeutic strategy against aggressive tumors.

## Abstract (web summary)

Stromal cells play various roles in tumor establishment and metastasis. Here the authors, using an ex-vivo model, show that cancer-associated fibroblasts facilitate colon cancer cells invasion in a matrix metalloproteinase-independent manner, likely by pulling and stretching the basement membrane to form gaps.

## Introduction

The basal surface of the epithelium is underlined by the basement membrane (BM), a thin and dense sheet-like structure. The BM is mainly composed of collagen IV and laminin networks produced by coordinated actions of epithelial cells and stromal fibroblasts^1–4^. It provides structural support to the epithelium, promotes cell adhesion, maintains cell polarity, and plays a role in tissue compartmentalization by separating the epithelium from the stroma^2, 5^.

In localized tumors, at the stage of carcinoma in situ, the BM represents a physical barrier that prevents spreading of the primary tumor to adjacent tissues^5^. Thus, when carcinomas become invasive, the BM must be breached to allow cancer cells to escape. Cancer cells can perforate the BM using matrix metalloproteinases (MMP)-rich protrusions, called invadopodia^6–8^. However, stromal cells could contribute to this process, as they also produce matrix proteases^9^. Indeed, as the tumor progresses, the surrounding microenvironment evolves, becoming enriched in cancer-associated fibroblasts (CAFs), immune cells, blood vessels, and extracellular matrix (ECM)^10, 11^. It is now established that CAFs play a role in tumor formation, progression, and metastasis^9, 12–16^. For instance, an in vitro model of cancer cell invasion in the stroma shows that CAFs lead cancer cell invasion by making passageways through collagen I/Matrigel gels^17^. In addition, recently it has been shown that CAFs exert a physical force on cancer cells via heterotypical cell–cell interactions that stimulates their invasion^18^. However, it remains unknown whether CAFs cooperate with cancer cells at an earlier step, to breach the BM and trigger the transition from carcinoma in situ to an invasive stage.

Here we show that CAFs isolated from colon cancer patients promote cancer cell invasion through a mesenteric BM. In the presence of CAFs, cancer cells invade the BM in a MMP-independent manner. Instead, they actively remodel the BM by pulling, stretching, and softening the BM. We propose that in addition to proteolysis, mechanical forces exerted by CAFs represent an alternative mechanism of BM breaching.

## Results

### CAFs stimulate cancer cell invasion through the BM

**Fig. 1:**
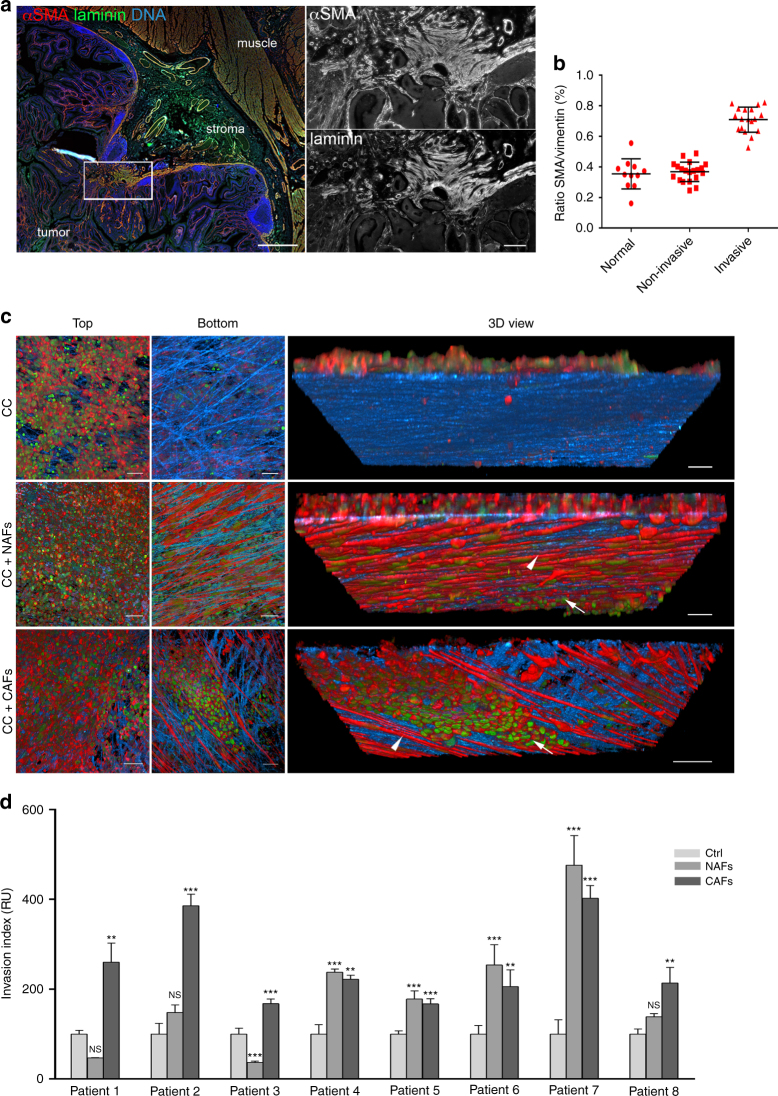
CAFs stimulate cancer cell invasion through the basement membrane. **a** Human colon carcinoma in situ. Basement membrane visualized by laminin staining (green), CAFs with αSMA (red), and DNA (DAPI, blue). Scale bar, 1000 µm. Boxed region was magnified; Invasive area showing accumulation of CAFs, and disorganization of the basement membrane. Scale bar, 200 µm. **b** Quantification of proportion of CAFs in human colon tissues: adjacent to the tumor (normal), non-invasive, and invasive carcinoma. Area occupied by CAFs was calculated as a ratio between αSMA and vimentin staining. For each patient five different areas were averaged. *p < 0.001, ANOVA, Krusskal–Wallis methods. **c** HCT116 colon cancer cells (CC) were cultured atop mouse mesenteric BM (top view) for 10 days, either alone or in the presence of NAFs or CAFs embedded in type I collagen matrix on the other side of the mesentery (bottom view). 3D view shows bottom part of the mesentery. Cells visualized by staining the actin cytoskeleton (phalloidin, red) and DNA (DAPI, green). The mesentery is detected by reflection (blue). Arrows show cancer cells and arrow heads show CAFs. Scale bars, 20 µm. **d** Quantification of cancer cell invasion through the mesenteric BM in the presence of NAFs or CAFs from eight different patients. The invasion index was calculated as the number of cancer cells per field on the underside of the BM. NAFs and CAFs from each patient are normalized to their respective controls. For each patient-derived CAFs and NAFs, and their respected controls about *n* = 5–12 fields were scored from one or two independent experiments. Mean ± s.e.m.***p < 0.0001; **p < 0.001; *p < 0.05; ANOVA, Dunn’s, or Holm–Sidak methods

Staining human colon carcinoma in situ samples for BM (laminin) and CAFs (αSMA) revealed a several layers thick capsule of αSMA (smooth muscle actin)-positive cells around the tumor, co-localizing with intact and continuous BM (Fig. 1a; Supplementary Fig. 1). Areas enriched with αSMA-positive cells coincided with displaced and discontinuous BM, suggesting that those cells could play a role in BM invasion. Using a cohort of human colon cancers of different stages, we found that αSMA-positive cells (generally called CAFs) were enriched in invasive tumors when compared to benign tumors or normal tissues lying adjacent to tumors (Fig. 1b).

The transient nature of carcinoma in situ makes the onset of cell invasion and BM breakage laborious to study in vivo. We therefore developed a 3D in vitro model that recapitulates the complexity of carcinoma in situ^19^. We isolated human fibroblasts from primary colon tumor specimens of eight patients (Supplementary Fig. 2a). For each patient, we obtained fibroblasts from the tumor mass (CAFs) and the juxta-tumoral tissue (NAFs; non-CAFs) (Supplementary Fig. 2b). Isolated cells were assessed for purity, characterized using known CAF markers and used as non-immortalized cells for up to 10 passages for further experiments (Supplementary Fig. 2c–e). As a model system of the BM, we used de-cellularized mouse mesentery (Supplementary Figs. 3 and 4), which recapitulates BM organization and characteristics more faithfully than in vitro polymerized gels such as Matrigel^6, 7, 19, 20^. To mimic carcinoma in situ, we plated cancer cells on one side of the mesentery, and CAFs embedded in type I collagen on the other side (Supplementary Fig. 5a). After several days of co-culture, cancer cells preserved a typical epithelial morphology, but CAFs adopted an elongated shape similar to that observed in vivo (Supplementary Fig. 5b). As mesentery properties vary between mice, experimental controls were always performed on mesenteries isolated from the same animal.

To quantify invasion, we counted the number of cancer cells found in the fibroblast compartment. We first checked if fibroblasts could stimulate invasion of early stage cancer cells. Neither NAFs nor CAFs were able to induce invasion of intrinsically non-invasive HT29 human colon cancer cells^7^, even after 25 days of co-culture (Supplementary Fig. 5b; Supplementary Movie 1). In contrast, CAFs augmented the invasion capacity of moderately invasive HCT116 colon cancer cells, suggesting a synergistic role for CAFs in cancer invasion and metastasis (Fig. 1c). Cancer cells were mostly invading collectively (Fig. 1c; Supplementary Movie 2), but small clusters and individual cells were also observed. While CAFs from all eight patients increased the invasion capacity of cancer cells, NAFs had diverse effects (Fig. 1d). NAFs from 50% of patients behaved similarly to their paired CAFs, while others were unable to stimulate cancer cell invasion. Interestingly, NAFs also showed heterogeneous expression of CAF markers, such as (αSMA) and seprase (FAP) (Supplementary Fig. 2). This suggests that juxta-tumoral regions that appear normal macroscopically can contain activated fibroblasts able to promote cancer cell invasion.

### CAFs remodel the BM and stimulate cancer cell invasion

**Fig. 2:**
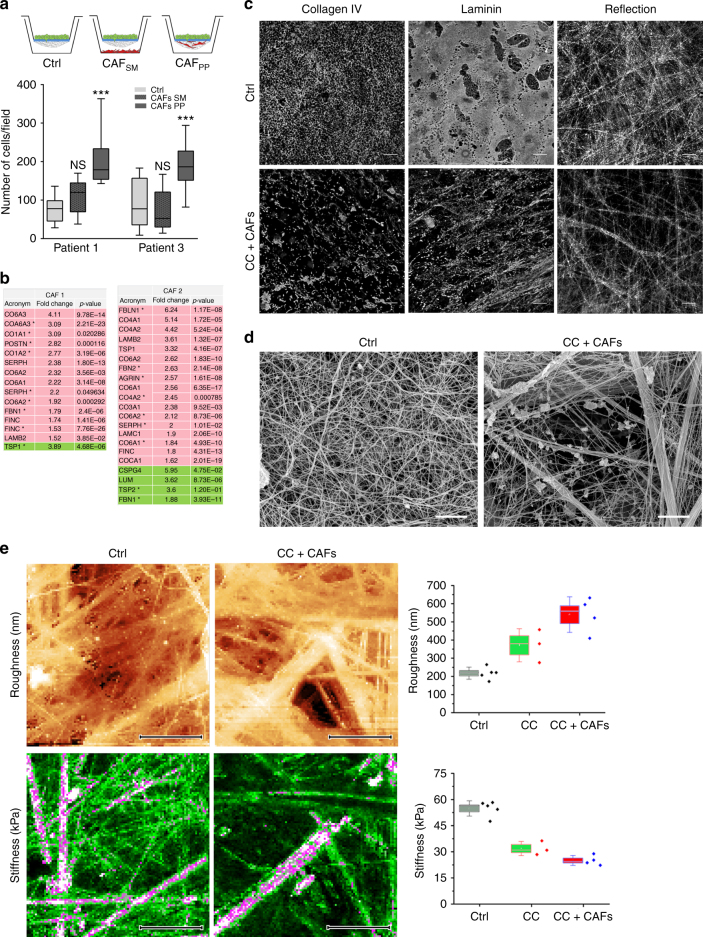
CAFs remodel the BM and stimulate cancer cell invasion. **a** Quantification of cancer cell invasion through mesenteric BM in the physical presence of CAFs (CAF PP) or their secreted molecules (CAF SM) from patients 1 and 3. Data are represented as box and whiskers (10–90 percentile) plus outliers. Data are presented from two independent experiments, *n* = 12 fields per condition. ***p < 0.0001; ANOVA, Dunn method used. **b** Manually selected list of ECM-related proteins present in higher (pink) or lower (green) amounts in CAFs from patients 1 and 2 compared with their paired NAFs. For each protein, fold change and *p*-value are presented. Peptide ratios with a *p*-value ≤ 0.05 are reported as significant. **c** Comparison of non-treated mesentery (Ctrl) and mesentery treated with HCT116 cancer cells and CAFs (CC + CAFs). Collagen IV and laminin networks were revealed using specific antibodies. ECM organization was evaluated by reflection microscopy. Scale bars, 20 µm. **d** Scanning electron micrographs showing non-treated mesentery (Ctrl) and mesentery treated with cancer cells and CAFs from patient 3 (CC + CAFs). Scale bars, 2 µm. **e** (Top) AFM quasi-height maps show roughness of non-treated mesentery (Ctrl) (color scale = 1.2 µm) and mesentery treated with cancer cells and CAFs from patient 3 (CC + CAFs) (color scale = 2.4 µm). Maps are 30 µm × 15 µm, *n* = 3–5 maps on one mesentery per condition from three independent experiments, dagger = *p* < 0.01, double dagger = *p* < 0.005, paired Student’s *t* test. (Bottom) AFM stiffness maps display sparse stiff fibers and many fibers with intermediate stiffness for the Ctrl condition, while in the CC + CAF condition, only few thick fibers appear, interspersed through an inhomogeneous soft mass. Color scales = 20–250 kPa, Maps are 30 µm × 15 µm, *n* = 3–5 maps on one mesentery per condition from three independent experiments. Dagger = *p* < 0.01, double dagger = *p* < 0.005, paired Student’s *t* test

We next investigated whether the physical contact between CAFs and the BM was necessary to stimulate invasion, or whether diffusible molecules secreted by CAFs were sufficient to increase cancer cells’ invasive capacity (Fig. 2a). We found that if the physical contact between CAFs and the BM was eliminated by placing CAFs in distance, while still allowing biochemical crosstalk between the two cell populations, cancer cells were unable to invade (Fig. 2a). Furthermore, Affymetrix arrays revealed no major differences in the expression of known invasion-related genes in these cancer cells, even though several signaling pathways were affected (Supplementary Data 1–4). These results indicate that physical contact between CAFs and the BM is required to stimulate the invasion of cancer cells.

To investigate how CAFs could stimulate cancer cell invasion, we compared the expression patterns of CAFs and NAFs from patients 1 and 2 using a proteomic approach, namely stable isotope labeling by amino acids in cell culture (SILAC) (Supplementary Data 5–12). We first observed that there was a limited overlap between the two patients in differently expressed proteins in CAFs compared with NAFs, pointing to the high heterogeneity of fibroblast populations (Supplementary Fig. 6). However, the proteomic analysis showed that CAFs from both patients produced higher amounts of ECM components and matrix remodeling proteins compared with their paired NAFs (Fig. 2b), as previously shown in other tumors^16^. We then compared the organization of laminin and collagen IV networks in mesenteries before and after culture with CAFs (Fig. 2c). Although cancer cells alone remodeled one of the laminin layers, the remodeling of laminin and collagen IV networks was more pronounced in the presence of CAFs. CAFs also reduced the BM reflection intensity signal by 30% (*n* = 7 mesenteries), which suggested that the general composition/organization of the ECM networks was altered. Indeed, using scanning electron microscopy (SEM) on the mesenteries remodeled by cancer cells and CAFs, we observed frequent holes often surrounded by big bundles of the ECM that were absent in control BM (Supplementary Fig. 2d).

To test the physical properties of remodeled BM, we measured BM stiffness under different conditions using atomic force microscopy (AFM). Compared with control BM, BM modified by cancer cells and CAFs was significantly softer (Fig. 2e). It showed a marked reduction in fibers of intermediate stiffness, consisting instead of patches of soft, inhomogeneous material sparsely interspersed with thick fibers, which also increased the overall roughness of the BM (Fig. 2e). These data reveal that the decrease in BM stiffness is most likely the result of rearrangements of ECM networks, as CAFs alter the molecular composition, organization, and physical properties of the BM, making it permissive to cancer cell invasion.

### CAFs-induced invasion of cancer cells is MMP-independent

**Fig. 3:**
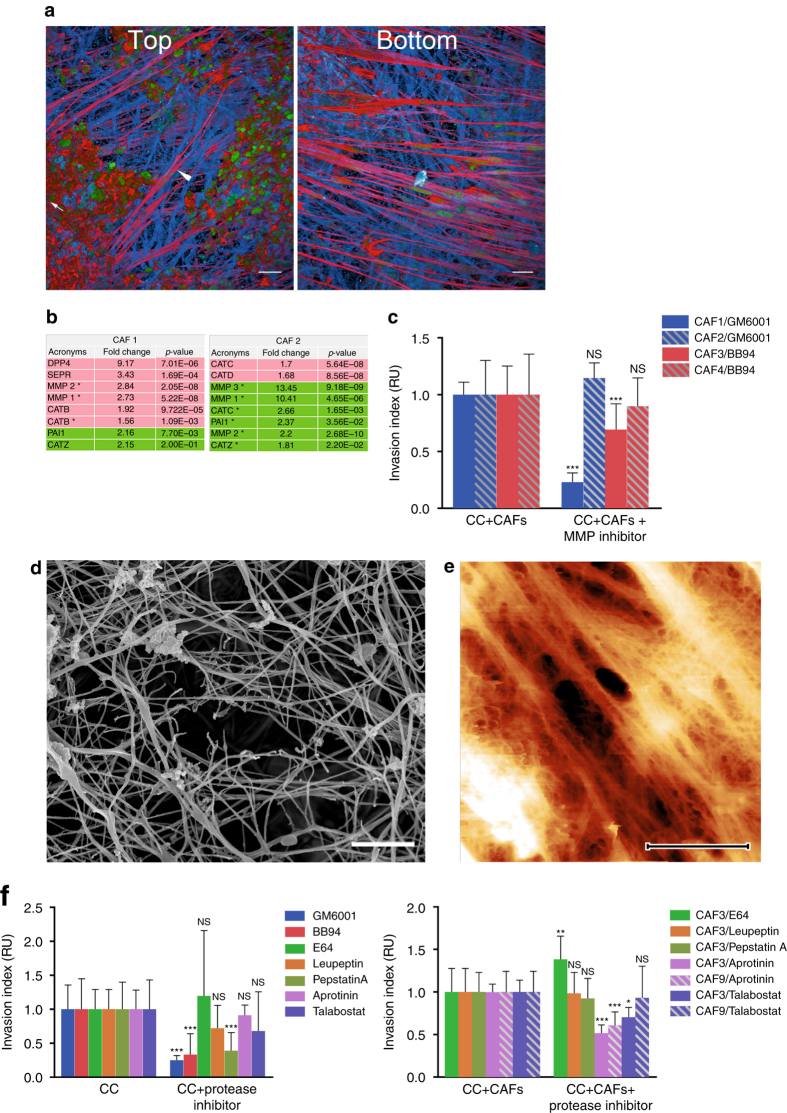
CAFs can stimulate cancer cell invasion in an MMP-independent manner. **a** HCT116 cancer cells co-cultured with CAFs on mouse mesentery for 10 days. Top view: CAFs (arrow head) breached the BM and migrated into the cancer cell (arrow) compartment. Bottom view: fibroblast compartment. Cells visualized by staining of the F-actin cytoskeleton (phalloidin, red) and DNA (DAPI, green). The basement membrane detected by reflection (blue). Scale bars, 20 µm. **b** Manually selected list of proteins with protease activity present in higher (pink) or lower (green) amounts in CAFs from patients 1 or 2 compared to their paired counterparts NAFs. For each protein, fold change and *p*-value are presented. **c** Quantification of cancer cell invasion through mesenteric BM in the presence of different drugs. For MMP inhibitor GM6001 and CAFs from patients 1 and 2, data from one experiment (*n* = 5–8 per condition) are presented. For MMP inhibitor BB94 and CAFs from patients 3 (*n* = 13–17 per condition) and 4 (*n* = 10–11 per condition), data are presented from three (patient 3) and two (patient 4) independent experiments. All conditions are reported to their respective controls. Mean ± s.e.m. ***p < 0.0001; **p < 0.001; *p < 0.05; pANOVA, Holm–Sidak method. **d** Scanning electron micrograph of cancer cell and CAF-modified BM in the presence of 10 µM BB94. Scale bar: 2 µm. **e** AFM quasi-height maps show roughness of mesentery cultured with cancer cells and CAFs, and treated with BB94. **f** Quantification of HCT116 cancer cell invasion through mesenteric BM in the presence of different drugs. Left: invasion of cancer cells cultured alone. Right: cancer cells cultured with CAFs. Data are presented from two independent experiments for Pepstatin A, Aprotinin, and Talabostat and three independent experiments for BB94. All conditions are reported to their respective controls. Mean ± s.e.m. ***p < 0.0001; **p < 0.001; *p < 0.05; pANOVA, Holm–Sidak method

One of the mechanisms by which CAFs could facilitate cancer cell invasion would be by digesting components of the BM, creating holes through which cancer cells could squeeze. Indeed, we occasionally observed CAFs on top of the BM even in regions where cancer cells had not invaded, suggesting that CAFs can breach the BM and translocate to the other side (Fig. 3a; Supplementary Fig. 5). Thus, we compared the production of proteases in CAFs and NAFs by SILAC (Fig. 3b). Interestingly, while CAFs from both patients increased cancer cell invasion as opposed to their respective NAFs, CAFs from patient 1 produced higher levels of MMPs, while CAFs from patient 2 showed reduced expression of MMPs (Fig. 3b). This suggests that the invasion capacity of CAFs is most likely not dependent on MMPs.

To directly test if cancer cell invasion is dependent on MMP activity, we used two general MMP inhibitors, GM6001 and BB94. When HCT116 cancer cells were cultured alone on the BM, their invasion was inhibited in the presence of those inhibitors, as previously reported for other cancer types^6, 7^ (Fig. 3f). Although protease inhibition significantly reduced cancer cell invasion in the presence of CAFs from patient 1, in the presence of CAFs from patients 2, 3, and 4 cancer cell invasion was not inhibited by GM6001 or BB94, pointing to an MMP-independent invasion (Fig. 3c). This was additionally supported by the electron microscopy and AFM imaging data, which revealed the presence of holes in the BM even with MMPs inhibited (Fig. 3d, e). Although MMPs are the only proteases involved in invasion of cancer cells through the BM^21^, it is possible that CAFs use other matrix proteases such as serine-, aspartyl-, or cysteinyl-proteases to help cancer cell invasion. To address this possibility, we used broad-spectrum protease inhibitors such as E-64, Leupeptin, Pepstatin A, Aprotinin, and Talabostat, targeting a wide range of matrix proteases, including cathepsins, urokinase-type plasminogen activator (uPA), and FAP^21, 22, 23^. While most of those inhibitors did not affect invasion of cancer cells, aprotinin decreased but did not block invasion of cancer cells in the presence of CAFs from two different patients, suggesting that uPA could be involved in the invasion of cancer cells through the BM (Fig. 3f).

Taken together, our findings revealed that all CAFs isolated from different patients stimulate cancer cell invasion, however they use most likely different mechanisms.

### CAFs stimulate invasion of cancer cells by remodeling BM

**Fig. 4:**
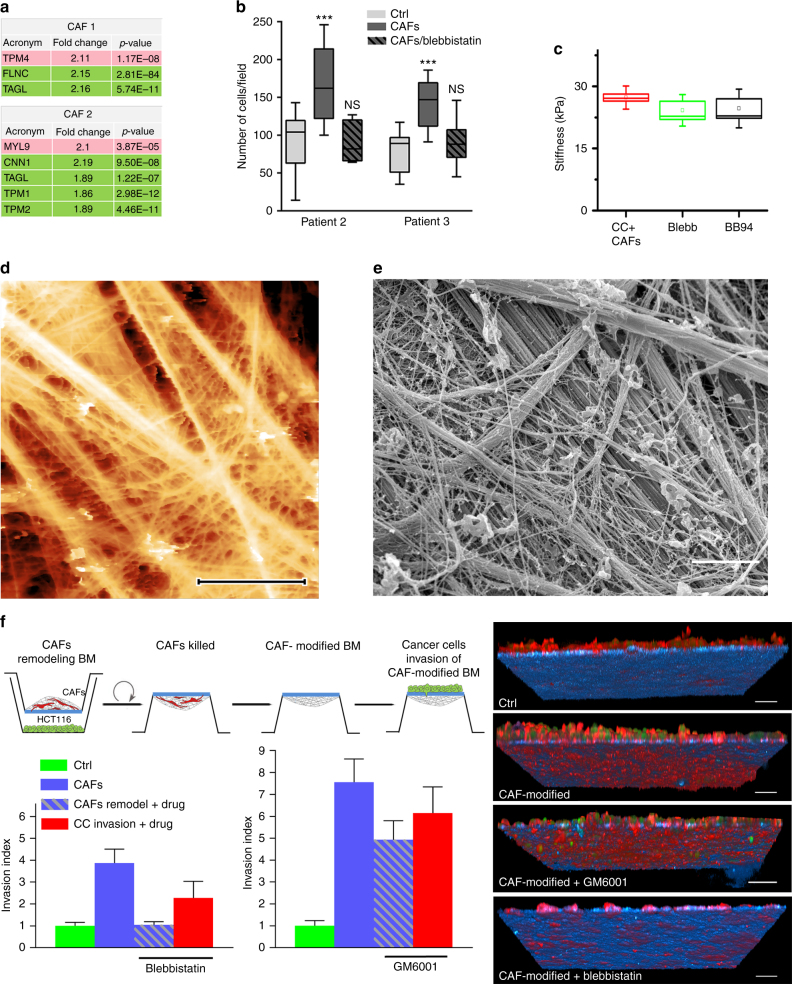
CAFs contractility is crucial to remodel the BM and make it permissive for cancer cell invasion. **a** Manually selected list of contractility-related proteins present in higher (pink) or lower (green) amounts in CAFs from patients 1 and 2 compared with their paired NAFs. For each protein, fold change and *p*-value are presented. Peptide ratios with a *p*-value ≤0.05 are reported as significant ratios. **b** Quantification of cancer cell invasion through the mesenteric BM in the presence of CAFs from patients 2 and 3 and the myosin II inhibitor Blebbistatin. Data are presented from two independent experiments (*n* = 11–13 fields per condition). Mean ± s.e.m. ***p < 0.0001; ANOVA, Holm–Sidak method. **c** Quantification of stiffness mesenteries remodeled by HCT116 cancer cells and CAFs extracted from patient 3. AFM maps are 30 µm × 15 µm, *n* = 3–5 maps on one mesentery per condition from three independent experiments. Dagger = *p* < 0.01, double dagger = *p* < 0.005, paired Student’s *t* test. **d** AFM quasi-height maps show roughness of mesentery cultured with cancer cells and CAFs, and treated with Blebbistatin. **e** Scanning electron micrograph of cancer cells and CAF-modified BM in the presence of Blebbistatin. Scale bar: 2 µm. **f** Invasion of cancer cells through CAF-modified BMs. BMs were modified with CAFs 2 for 7 days in the distant presence of cancer cells. CAFs were then killed to generate CAF-modified BM on which cancer cells were cultured for 5 days. GM6001 and Blebbistatin were added either during the CAF remodeling phase or during the cancer cell invasion phase. Right: 3D view showing cancer cells that have invaded mesentery in different conditions. Cells visualized by staining the actin cytoskeleton (phalloidin, red) and DNA (DAPI, green). Mesentery detected by reflection (blue). Scale bars: 20 µm. Left: quantification of cancer cell invasion of the non-modified (Ctrl) and CAF-modified BM. Blebbistatin or GM6001 were added either during the CAF remodeling phase (CAFs remodeling + drug) or during the cancer cell invasion phase (CC invasion + drug). Invasion of cancer cells normalized to Ctrl. *N* = 1–2, *n* = 5–13 per condition. Mean ± s.e.m. *p < 0.05; ANOVA, Dunn’s method

Because matrix proteolysis was not necessary for all CAF-driven cancer cell invasion, we examined alternative mechanisms that CAFs could use to enhance BM invasion. Our proteomic analysis showed that, compared with their paired NAFs, CAFs expressed increased levels of proteins associated with high contractility, such as myosin light chain (MYL9) and tropomyosin 4 (TRP4), and lower levels of proteins associated with reduced contractility, such as tropomyosin 1 and 2 (TPM1 and 2), filamin-C (FINC), transgelin (TAGL), and calponin (CNN) (Fig. 4a; Supplementary Table 2). Some of those proteins were previously found in CAFs isolated from different types of tumors^13, 24^. As expected, CAFs exhibited an increased capacity to contract 3D collagen gels compared to NAFs (Supplementary Fig. 7) and expressed higher levels of αSMA, which correlates with higher contractility^25^ (Supplementary Fig. 2; Supplementary Table 2). To test if CAF contractility facilitates cancer cell invasion in our system, we treated co-cultures with the myosin II inhibitor blebbistatin. Inhibition of actomyosin contractility reduced cancer cell invasion through the BM (Fig. 4b). Interestingly, even though blebbistatin treatment did not affect BM softening (Fig. 4c), the previously evident holes in the BM were not apparent (Fig. 4d, e). These results suggest that cancer cells and CAFs inability to contract prohibits BM remodeling to an extent that hinders cancer cell invasion.

Next, we asked whether BM remodeling by CAFs makes it more permissive for cancer cell invasion. To test this, we evaluated the capacity of cancer cells to invade BM on which CAFs had previously been cultured (Fig. 4f). In this setup, we allowed the crosstalk between CAFs and cancer cells, but only CAFs could physically interact with the BM. We then eliminated CAFs and cultured cancer cells on top of this CAF-modified BM (Fig. 4f). While only a few cancer cells invaded non-modified BM (Ctrl) after 5 days of culture, at least four times as many invaded the CAF-modified BM (Fig. 4f). CAF-mediated remodeling of the BM is therefore sufficient to promote cancer cell invasion. Interestingly, cancer cells treated with MMP inhibitors retained their ability to invade CAF-modified BM (Fig. 4f), and were also able to invade BMs remodeled by MMP inhibitor-treated CAFs, confirming that BM remodeling occurs via an MMP-independent mechanism. In contrast, if actomyosin contractility in CAFs was inhibited during their BM remodeling phase, cancer cells were no longer able to invade the BM. As expected, when cancer cells were treated with blebbistatin, they lost their ability to invade CAF-modified BM because cell contractility inherently also regulates cell migration^26^.

These results indicate that CAFs stimulate cancer cell invasion indirectly, by applying mechanical forces on the BM, thereby remodeling the BM and making it permissive for cancer cell invasion.

### CAFs use physical forces to remodel the BM

**Fig. 5:**
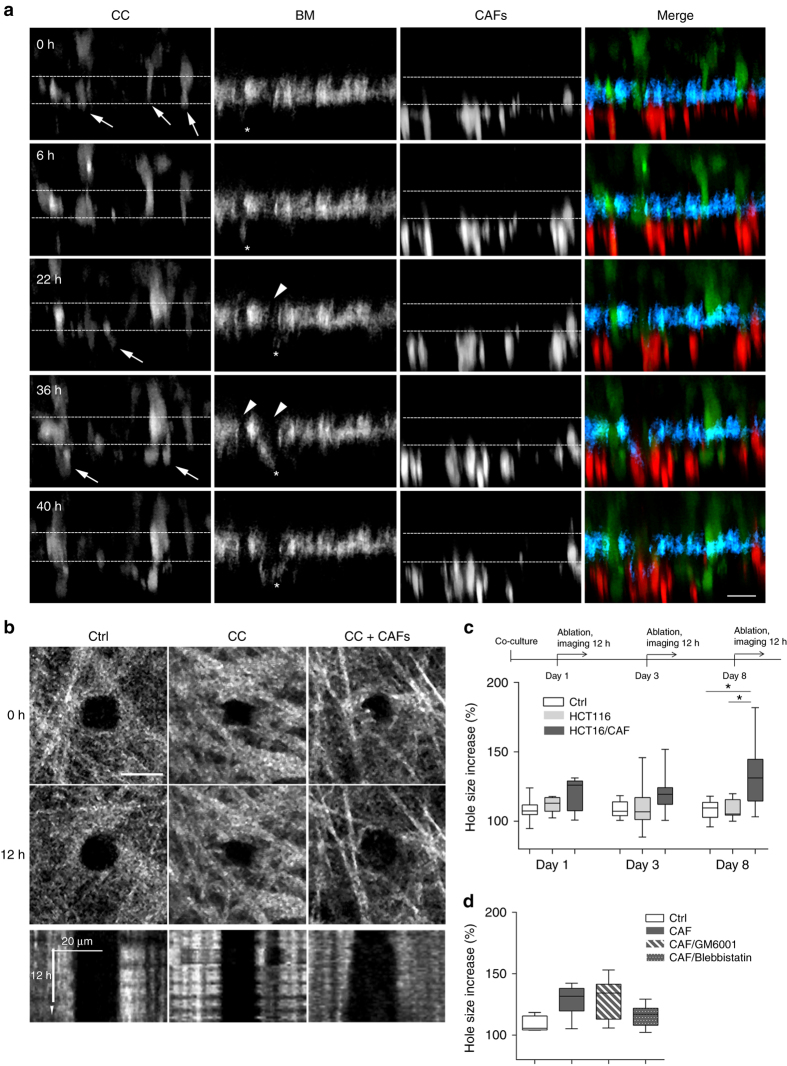
CAFs exert physical forces on the BM to stimulate cancer cell invasion. **a** Time-lapse analysis of BM invasion. *y/z* resliced images of co-cultures of cancer cells expressing cytoplasmic GFP (green) and CAFs labeled by vital dye (red) over time (in hours). Mesentery revealed by reflection (cyan). Scale bars, 20 µm. Imaging started at 5 days of co-culture. Arrow head points to gaps in the BM. Arrows indicate cancer cell protrusions that extend into the BM and cancer cells translocated on the other side of the BM. Asterisks indicate CAFs that pull the BM. Dashed line guides for eye representing the BM borders. **b** CAFs widen already existing holes in the mesentery. Top: *x/y* projections of mouse mesentery revealed by reflection microscopy. Mesenteries were cultured for 3 days either without cells (Ctrl), with cancer cells, or with both cancer cells and CAFs 2. 100 µm^2^ holes were created in the mesentery using laser ablation at time 0. The size of the hole was followed over a period of 12 h. Scale bars, 20 µm. Bottom: kymograph showing the size of the hole over 12 h. **c** Increase in gap size after 12 h relative to the initial size of the hole at the beginning of the experiment performed on the control mesentery without any cells (Ctrl), cultured with cancer cells alone or with cancer cells and CAFs from patient 2. The ablation and measurements are taken after 1, 3, or 8 days of culture. Data from one (1 day culture) and two (3 and 8 days culture) independent experiments are presented, *n* = 5–14 per condition; *p < 0.05; ANOVA, Dunn’s method used. **d** Increase of the hole size after 12 h performed on the mesentery cultured with cancer cells and CAFs from patient 2 for 8 days in the presence of GM6001 and Blebbistatin, *n* = 4–7 fields per condition from one experiment. *p < 0.05; ANOVA, Shapiro–Wilk method used

To explore how CAFs mechanically induce cancer cell invasion, we performed long-term live imaging of our BM setup (Supplementary Movie 3). At early time points (3 days of co-culture), both cancer cells and CAFs migrated in an uncoordinated manner along the *x/y* plane, but remained on their respective sides of the BM (Supplementary Fig. 8a; Supplementary Movies 4, 5). For each 100 µm of BM, cancer cells extended on average 1.1 ± 0.4 (mean ± s.d.) protrusions that passed through the BM and made contact with CAFs present on the other side. The protrusions became more frequent with time (2.4 ± 0.4 protrusions/100 µm at 4 days). After 5 days of co-culture, CAFs adopted an elongated, needle-like shape and coordinated their movement (Supplementary Movie 6). From Day 6, cancer cells translocated to the CAF side (Fig. 5a; Supplementary Movie 7). Interestingly, transient but frequent gaps in the BM formed at this time point (0.6 holes/100 µm in 45 h). CAFs were present in close proximity to these gaps, suggesting a role for CAFs in gap formation. Indeed, we observed CAFs pulling the BM toward them, possibly stretching small, pre-existing gaps. When stretched to their maxima, these gaps were on average 6.2 ± 1.7 µm in diameter (mean ± s.d., ranging from 3.4 to 12.8 µm, *n* = 95), wide enough for cancer cells to squeeze through^27, 28^.

To investigate whether CAFs could widen pre-existing gaps, as suggested by our previous observation, we seeded cancer cells either alone or together with CAFs on the BM. We then applied laser ablation to the mesentery to create 10 × 10 µm holes (Fig. 5b; Supplementary Movie 8). Gap size remained unchanged over 12 h if the BM was cultured without any cells or only with cancer cells. In the presence of both cancer cells and CAFs, however, the holes grew larger (Fig. 3b, c). To see whether cells required time to remodel the BM more efficiently, we pre-cultured mesenteries with both cancer cells and CAFs for 1, 3, or 8 days before creating holes. The holes generated in the “naked” BMs or BMs covered with cancer cells did not significantly increase in size over time (Fig. 5c). In contrast, the longer the cancer cells and CAFs were co-cultured on the BM, the more pronounced the gap widening became (from 10 to 60% over time), suggesting that CAFs use a time-dependent mechanism to stretch the BM. We then asked whether the gap widening is driven by MMP proteolysis of the matrix or by mechanical stretching. Addition of GM6001 did not affect the widening of laser ablation-generated holes in the presence of CAFs (Fig. 5d). In contrast, inhibiting myosin II-driven contractility with blebbistatin inhibited widening. CAFs could therefore use their contractility to pull on BM fibers and physically widen the pores between them. Indeed, we frequently observed fiber separation in the presence of CAFs (Supplementary Fig. 8b). Taken together, these results indicate that CAFs are able to widen the holes in the BM through an MMP-independent but contractility-dependent mechanism and facilitate cancer cell invasion.

## Discussion

Overall, our results demonstrate that CAFs actively assist cancer cells to breach the BM. Proteomic analysis of primary human CAFs showed that CAFs isolated from different patients are heterogeneous, confirming previous findings^13^. However, some CAF features could be identified, as CAFs from both patients expressed higher amounts of ECM components, matrix remodelers, and contractility-related proteins, compared to their paired NAFs. Remodeling of the BM by CAFs was critical to stimulate cancer cell invasion. However, reduction in BM stiffness and cancer cell invasion did not correlate linearly. While cancer cells and CAFs soften the BM in the presence of both inhibitors of MMPs and actomyosin contractility, invasion was inhibited only if contractility was blocked, suggesting that reduction of BM stiffness alone is not sufficient to stimulate invasion. However, scanning electron micrographs and AFM topography showed that only actomyosin inhibition prevents formation of holes in the BM. By contracting the BM, CAFs widen the pre-existing holes, which consequently stimulates cancer cell invasion. These pre-existing holes could be initially created by MMPs or other proteases such as uPA, or they could be an intrinsic property of the BM. For example, venular BMs contain low expression regions of laminin and collagen IV that leukocytes use to transmigrate^29^. Interestingly, BMs in mesenteries also contain laminin and collagen IV low expression regions (with an average area of 10.2±3.5 μm^2^ for laminin and 21.3±9.1 μm^2^ for collagen IV). It is therefore possible that cancer cells and CAFs start remodeling the BM from those “weak” regions. While deciphering the complete mechanism by which CAFs help cancer cells to breach the BM in vivo would require further investigation, our observations support the following model: At the carcinoma in situ stage, the tumor is encapsulated by the BM and a layer of CAFs. Even though cancer cells and CAFs are physically separated at this stage, they may physically interact through the BM. CAFs exert mechanical forces on the BM by pulling the ECM fibers and stretching pre-existing gaps. The disrupted organization of the ECM results in a decreased BM stiffness and integrity, thereby reducing its barrier functions and making it more permissive for invasion.

Normal developmental processes involving BM invasion also display cooperation between different cell types. In *Caenorhabditis elegans*, cells must cross the BM to unite the developing vulva and uterus. A specialized uterine cell, the anchor cell, forms an initial gap in the underlying BM, while vulval cells present on the opposite side of the BM promote sliding of the BM to expand this gap and help the anchor cell invade^5, 30^. This invasion is only partially dependent on MMPs; the moving vulval cells probably generate tensile forces that push the BM aside^31^. Physical forces also play a role in breaching the BM during post-implantation development in the mouse^32^, a process that probably does not require MMPs. Numerous studies show that cancer cells or other cells in the tumor microenvironment often hijack mechanisms used by normal cells during development. Our study shows that CAFs can use an MMP-independent mechanism to stimulate cancer cell invasion. This may explain why MMP inhibitors used as single drugs have failed in clinical trials^33, 34^. As an alternative to the protease-dependent invasion, cancer cells in synergy with stromal cells may adopt a strategy based on mechanical forces and physical remodeling of the tumor microenvironment. Blocking the ability of CAFs to contract and exert mechanical forces on the BM could therefore represent a new therapeutic strategy against spreading of aggressive tumors. Finally, it is crucial to investigate in the future if other cell types within the tumor microenvironment, such as tumor-associated macrophages, could assist cancer cells in breaching the BM, as they are known to promote tumor cell invasion through the stroma and intravasation^35^.

## Methods

### Antibodies

For immunofluorescence and/or western blot, we used: monoclonal anti-vimentin antibody purchased from Dako (clone V9) at a dilution of 1:100, monoclonal anti-α-tubulin (clone DM1A), rabbit anti-laminin, and monoclonal anti-α-SMA from Sigma-Aldrich and rabbit anti-collagen IV from Millipore at a dilution of 1:200, sheep anti-FAP from R&D Systems at a dilution of 1:50, and rabbit anti-α-SMA from Abcam at a dilution of 1:400. Rhodamine-labeled phalloidin and all secondary antibodies were purchased from Molecular Probes and were used at a dilution of 1:200. For flow cytometry we used: monoclonal APC Cy7 Mouse anti-Human CD45 antibody purchased from BD Biosciences (clone 2D1), monoclonal PerCP/Cy5.5 Mouse anti-Human CD326 (clone 9C4), and monoclonal PECy7 Mouse anti-Human CD31 (clone WM59) from Biolegend.

### Immunofluorescence and immunohistochemistry staining

Human colon tumors (20 adenomas and 18 adenocarcinomas) were obtained from the Gastroenterology and Pathology Unit of the Institut Curie, Paris. Tissues were fixed for 2 h AFA (ethanol: formalin: acetic acid at a ratio of 75:2:5) and paraffin-embedded. About 3 µm thick sections were cut and proceed for immunostaining. Antigen retrieval was performed for 20 min in a boiling antigen in unmasking solution (Vector Laboratories). Sections were blocked with 5% fetal calf serum in PBS, incubated with primary antibodies in the blocking solution for 2 h at room temperature (RT) or overnight at 4 °C, and followed by incubation with secondary antibodies for 1 h at RT. Sections were mounted in AquaPolymount (Polysciences). To estimate the enrichment of CAFs in the stroma, samples were stained with vimentin, a fibroblast marker and with *α*SMA, a CAFs marker. For each sample, images of five different fields of the tumor and one of the normal adjacent tissue were acquired using ×10 objective of an epifluorescence microscope (Leica DM6000B) equipped with a Cool Snap CCD camera. Images analysis was performed using Metamorph software (Roper scientific). Proportion of CAFs in stroma was calculated as a ratio between areas positive for *α*SMA (general marker of CAFs) and vimentin (label both CAFs and non-activated fibroblasts). Muscle tissue was not included while areas of αSMA staining around the vessels was subtracted from all images. For each patient, the values are presented as average of the five randomly chosen fields from the same sample.

Four carcinoma in situ were stained with Laminin/*α*SMA antibodies and whole slides were scanned with Pannoramic 250 Flash III (3D Histech, Hungary).

### Cell lines

Human colon cancer cells HT29 and HCT116 were obtained from American Type Culture Collection. All cells were cultured in DMEM medium supplemented with 10% FBS (Invitrogen) and 5% CO_2_.

### Extraction and culture of primary cells

Human primary fibroblasts were isolated from fresh colon tumors (CAFs) and adjacent healthy tissue (NAFs) samples from patients treated at Institut Curie Hospital, Paris, with written consent of the patients and approval of the local ethics committee. Samples were collected after surgical resection in Roswell Park Memorial Institute buffer (RPMI) and washed in phosphate buffered saline (PBS) supplemented with 1% Antibiotic-Antimycotic (Gibco). Fibroblasts were extracted as previously described^36^. Briefly, tissue was mechanically resected in 1 mm pieces and plated on scratched 10 cm Petri dishes. Cells were cultured in DMEM supplemented with 10% FBS and 1% Antibiotic-Antimycotic at 37 °C. Medium was changed every 3 days. Once emerged from tissue peace, fibroblasts were transferred in 30 kPa soft substrate plates (ExCellness Biotech SA). The soft plates were coated with collagen type I that was extracted from rat tails^19^. Collagen was diluted in DMEM serum-free medium at 5 µg/mL, applied on soft plates, and incubated at 37 °C for at least 48 h before plating cells. Cells were used up to 10 passages.

### Flow cytometry

Fibroblasts were detached from their support using 10 mM EDTA for 30 min at RT and re-suspended in Live/Dead Fixable Violet (Life Technologies) and incubated for 20 min at RT in the dark. Cells were washed twice with PBS, fixed with 4% PFA overnight at 4 °C, and washed twice with PBS supplemented with 5% of human serum (Biowest) and EDTA 2 mmol/L (Life Technologies). Cells were stained with the following antibodies: APCCy7-CD45, PerCP/Cy5.5-CD326, and PECy7-CD31. Flow cytometry analysis was performed with LSRFortessa flow cytometer (Becton Dickinson).

### Immunoblotting

Cells were washed with PBS on ice and immediately lysed in Laemmli sample buffer (62.5 mM Tris-HCl, pH 6.8, 25% glycerol, 2% SDS, 0.01% bromophenol blue). The samples were resolved by sodium dodecyl sulfate–polyacrylamide gel electrophoresis (SDS–PAGE) on 10% gels, transferred to nitrocellulose membrane, and blocked with blocking solution (5% non-fat dried milk in PBS supplemented with 0.1% Tween detergent) for 30 min. The membranes were incubated with primary antibodies for 1 h at RT or overnight at 4 °C, followed by incubation with Horseradish peroxidase-conjugated secondary antibodies for 1 h at RT. Immunoreactive bands were detected using an ECL-plus kit (Roche), and chemiluminescence detection was performed using either film or a BioRad ChemiDoc MP instrument.

### Contraction assay

About 1.5 × 10^5^ of fibroblasts were re-suspended in 1.5 mL of 2 mg/mL rat tail collagen I (BD) and added to a 24-well plate in triplicates (500 µL/well). After 30 min of incubation at RT, collagen plugs were detached from the walls of the well with a scalpel and DMEM supplemented with 10% FBS was added. Images of the collagen plugs were acquired at time 0 (T0) and after 24 h (T1) using a binocular microscope M165FC (Leica). The area of the plug was measured using ImageJ, and the plug contraction was presented as a ratio between the gel area *T* = 1 and *T* = 0 in %.

### Basement membrane isolation

All studies and procedures involving animals were in strict accordance with the European and National Regulation for the Protection of Vertebrate Animals used for Experimental and other Scientific Purposes (facility license #C75–05–18). Mesentery BM was isolated from 9 to 12 months old female C57/B6 mice. To avoid damage, mesentery was never handled directly. Holding on the mouse intestine with tweezers, the mesentery was placed and glued on the inserts of a 24-well plate 6.5 mm diameter transwell (BD Bioscience) (from which the polycarbonate membrane was removed) using chirurgical glue (3 M Vetbond). Mesentery was washed with cold PBS, incubated for 5 min in PBS supplemented with 1% Antibiotic-Antimycotic solution and then, to kill all resident mesothelial cells and strip the membrane, treated with 1 M ammonium hydroxide for 1 h at RT. Mesentery was washed three times with PBS and stored at 4 °C for up to 72 h. For a schematic representation of the protocol and more detailed information, we refer the reader to previous studies published by us and others^7, 19, 21^.

### Invasion assays

Collagen I (BD Biosciences) was diluted in 10× PBS to achieve 2.2 mg/mL. About 1 N of NaOH was added to adjust pH to 7.4. Fibroblasts were re-suspended in DMEM and mixed with collagen on ice to achieve a final cell density of 10^6^ cells/mL in 2 mg/mL collagen matrix. The holder with the treated mesentery was turned upside down and 80 µL of collagen (containing 8 × 10^4^ fibroblasts) was added on the mesentery (bottom) (Supplementary Fig. 5a). Once collagen was polymerized, the holder was turned and placed in a well filled with DMEM media supplemented with 10% FBS and 1% Antimycotic-Antibiotic. On the other side of the mesentery (top) 1.5 × 10^5^ tumor cells were plated. After 12–24 h, the medium was replaced with DMEM supplemented with 1% FBS and 1% Antimycotic-Antibiotic (complete medium). Cells were cultured between 3 and 25 days at 37 °C and 5% CO_2_. All experiments were run in complete medium (above), unless otherwise indicated, in the absence or presence of different drugs: 10 or 20 µM MMP inhibitor GM6001 (Calbiochem); 5 or 10 µM MMP inhibitor BB94 (Sigma-Aldrich); 200 µM Cysteine protease inhibitor E-64 (Sigma-Aldrich); 50 µM acid protease inhibitor Pepstatin A (Sigma-Aldrich); 250 µM Serine and cysteine protease inhibitor Leupeptin (Sigma-Aldrich); 200 µg/mL Serine protease inhibitor Aprotinin (Sigma-Aldrich); 10 µM Dipeptidyl peptidase inhibitor Talabostat; 17 or 20 µM Blebbistatin (Sigma-Aldrich). All drugs were added on both sides of mesentery with each medium change at days 1 and 5 of the experiment.

To examine molecular composition of a mesentery, mesenteries were prepared as described above, and then incubated at 37 °C and 5% CO_2_ for 7 days in the presence or absence of cells. Cells were killed by the addition of 10 µg/mL puromycin for 2 days. Mesentery was further treated with 1 M ammonium hydroxide for 40 min to remove cell debris and washed extensively in PBS before proceeding with immunofluorescence. The same results were obtained in three independent experiments.

For the invasion assay with CAFs-modified BM, 80 × 10^4^ fibroblasts in an 80 µL collagen type I drop were added on the holder with mesentery and collagen was polymerized at RT for 15 min. The holder was then placed in a well of a 24-well plate containing 1.5 × 10^5^ HCT116 cells in DMEM and 10% FBS. After 24 h the media was changed to DMEM with 1% FBS and incubated at 37 °C, 5% CO_2_. After 7 days, mesentery was washed with PBS, treated with 10 µg/mL puromycin for 2 days and washed extensively with serum-free DMEM. About 1.5 × 10^5^ HCT116 cells concentrated in a 50 µL DMEM were added on the bottom side of the BM (the opposite side to where the fibroblasts were). After 20 min cells adhered to the mesentery, and holders were placed in a 24-well plate well containing DMEM with 1% FBS and incubated at 37 °C, 5% CO_2_ for 5 days.

### Immunofluorescence

Cells were pre-extracted for 3 min in 0.5% Triton X-100 in PEM buffer (100 mM PIPES, pH 6.9; 1 mM MgCl_2_; 1 mM EGTA) and washed with PEM buffer. Cells were then fixed with 4% PFA for 40 min and washed three times for 10 min in PBS. Cells were incubated with primary antibodies overnight at 4 °C and then washed three times for 15 min at RT. In secondary antibodies, cells were incubated for 2 h at RT and then washed three times for 15 min. For all incubations and washing, the solutions were added on both sides of the membrane, and membranes were constantly kept hydrated. The sample was then mounted on a glass-bottom dish with Polymount medium (Polysciences) applied on both sides of the membrane. Samples were kept in the dark at 4 °C. To reveal F-actin and DNA, cells were stained with rhodamin-phaloidin and DAPI, respectively, added with the primary antibody incubation.

### Microscopy and imaging processing

Cells were imaged with a laser scanning confocal microscope LSM 710 NLO (Zeiss, Jena, Germany) coupled with Argon 488 laser (GFP), DPSS laser 561 (rhodamine), and diode 405 (DAPI) using 20×/0.8NA and 40×/1.2NA oil-immersion objectives (Zeiss). BM was visualized by reflectance confocal microscopy, using visible light at a wavelength of 488 nm and a standard photomultiplier tube (PMT) detector. 3D stacks were obtained at a step size of 1.35 µm intervals. The images were processed with ImageJ (NIH) or Imaris (Bitplane). Standard contrast and intensity levels were further adjusted linearly using Photoshop (Adobe). Invasion index was calculated as a number of cancer cells on the bottom side of the mesentery per field obtained with × 20 objective.

### Time-lapse microscopy

For time-lapse experiments, we have used 1.5 × 10^5^ HCT116 cancer cells expressing cytoplasmic GFP. About 0.8 × 10^5^ CAFs were stained with a lyophilic carbocyanine dye (Vybrant DiI-Cell labeling Solutions, Thermo Fisher) according to the manufacturer recommendations. Cells were plated on the mesentery as described above. The holder was placed on two glass-squared section capillaries 1 mm height and 1.5 cm length (VITROCELLS) that were glued on the glass-bottom dish using Loctite hysol glue. The capillaries were used as pillars in order to increase the space between the sample and the glass-bottom dish, to attain the working distance of the objective and avoid the acquisition of reflection of the glass bottom of the dish. In addition, this allowed medium to flow freely, so that both sides of the mesentery are immerged in medium. The dish was incubated at 5% CO_2_, 37 °C in the on-stage incubator (Okolab). Images were acquired with an inverted AOBS two-photon laser scanning confocal microscope SP8 (Leica) coupled to femtosecond laser Chameleon Vision II, Ti:Sapphire pumped Optical Parametric Oscillator (680–1400 nm) (Coherent Inc) using 25×/1.0NA water-immersion objective. The microscope is equipped with two non-descanned detectors: NDD1 (500–550 nm), NDD2 (≥590 nm). Fluorescence channels were recorded simultaneously using the excitation wavelength 960 nm. BM was visualized by reflection microscopy, using light at a wavelength of 488 nm and a standard PMT detector, at a low gain (value of 500 in a Leica SP8). Images were recorded every 15 min up to 72 h. 3D stacks were obtained at a step size of 1 µm intervals. Data are analyzed from 1–2 experiments. The images were processed with Leica Application Suite (LAS), ImageJ (NIH), or Imaris (Bitplane). Standard contrast and intensity levels were further adjusted using Photoshop (Adobe).

### Laser ablation

Samples were prepared as described above for the invasion assay and incubated at 37 °C, 5% CO_2_. Two-photon SP8 confocal microscope (Leica) laser at a wavelength of 800 nm was used to make 100 μm^2^ holes. To make holes only in the mesentery without damaging the collagen network underneath, the beam was focused on the mesentery that has characteristic reflection appearance. The entire mesentery was ablated using the maximal power of the laser for 3 s at the bidirectional scan speed of 800 Hz, and the focal plane was moved manually in the opposite direction of the collagen gel. For each sample, 5 ablations at least 100 µm apart were performed. Time-lapse imaging of the holes started right after the ablation and run for 12 h, with a 15 min time step. Images were processed in LAS AF Lite software, ImageJ, and Imaris. The size of the gap was measured at each time point using ImageJ. Increase in hole size was presented as a ratio between the size of the hole at *t* = 0 and *t* = 12 h in %.

### Scanning electron microscopy

Sample preparation: BM samples were fixed in 2.5% glutaraldehyde solution, in a 0.1 M cacodylate buffer overnight, at 4 °C. They were then washed three times in a 0.2 M cacodylate buffer and post-fixed for 30 min at RT with 2% osmium tetroxide in 0.2 M cacodylate buffer. Samples were then dehydrated in a series of graded ethanol baths (25, 50, 75, 95, and 100%) and then transferred to the SEM lab where they were treated for critical point drying by CO_2_, using Baltec CPD030 technology and gold–palladium metallization, using an Ion Beam Coater GATAN. Observations were made with a Jeol 6700F microscope in Pasteur Institute (Paris, France).

### Atomic force microscopy

Sample preparation: Mesentery stringed culture inserts were placed onto glass slides pre-incubated with poly-l-lysin solution for 10 min. The de-cellularized membrane was facing the glass slide. The insert with the glass slide was centrifuged for 10 min at 3000 rcf prior to insert being carefully cut off from the membrane with a razor blade, leaving the membrane firmly attached on the glass slide and stored in PBS at 4 °C until further use. For stiffness measurements of cells under influence of drugs, cells were seeded onto TPP culture dishes at a density of 100,000 cells/cm^2^ in DMEM with Glutamax supplemented with 1% FBS. After 24 h, BB94 (10 µM) or Blebbistatin (20 µM) were added to DMEM supplemented with 1% FBS and the medium replenished. The medium was changed at Day 4 and consequently AFM force mapping was performed as described below.

### AFM force mapping

The AFM was comprised of a Nanonis AFM Controller (SPECS Zurich GmBH, Switzerland) using a custom written software. For all measurements of mesenteries, DNP-10 D (nominal stiffness 60 mN/m) cantilevers (Bruker AFM Probes, USA) were used. For measurements of cells, HQ-CSC38/CrAu B (nominal stiffness 30 mN/m) cantilevers (MikroMasch, Nanoworld AG, Switzerland) were used. Additional roughness measurements of mesenteries treated with either BB94 or Blebbistatin were performed with Nanowizard 4 (JPK, Germany) using QI mode and SCANASYST-FLUID (Bruker) cantilevers having a nominal spring constant of 0.35 N/m. Cantilever stiffness was calibrated in air using the Sader method prior to experimentation. Glass slides with mesentery BMs or dishes with cells were mounted on the combined AFM and inverted microscope (Zeiss Axio-Observer A1, Germany) setup. Low-resolution force maps for stiffness evaluation of mesentery were recorded at 60 × 60 µm, and 32 × 32 force curve (pixel) resolution. High-resolution force maps for roughness evaluation were measured at 30 × 30 µm and 100 × 100 force curve (pixel) resolution. All maps on mesentery were measured at a maximum loading force of 3.1 nN and indentation velocity of 16 µm/s. Cell stiffness was assessed with force maps recorded at 32 × 32 pixels and 40 × 40 µm at a maximum loading force of 1.8 nN and indentation velocity of 16 µm/s. QI images were obtained at 30 × 30 µm and 512 × 512 pixel with a loading force of 10 nN.

### AFM data analysis

Force maps were analyzed using the custom-made software in Labview. All curves were transformed to force vs. tip-sample distance. First the tilt and offset were corrected. Subsequently the contact area was fitted using a power law (power = 2) and the non-contact area with a straight line. The E-modulus was calculated from the unloading curve and according to the modified Oliver-Pharr model (Plodinec and Lim^37^). For roughness analysis, quasi-topography data were extracted from contact points measured during force mapping. QI imaging mode provided the topography data. These were then imported into Gwyddion, where a plane level fit was performed and the RMS of the area was used to quantify the surface roughness of mesenteries. For the high-resolution data, in total 12 maps were generated each comprising 10,000 force curves. Mesentery data were analyzed in least three independent experiments (for high-resolution, counting one mesentery as one experiment) and six low-resolution experiments (two mesenteries for each condition) from which the representative mesenteries were used for high-resolution testing). The nanomechanical measurements of HCT16 cells were performed on minimum three different culture dishes per condition and by measuring at least four maps per condition.

### Proteomic analysis of CAFs and NAFs

To prepare SILAC media, SILAC DMEM lacking two amino acids, arginine and lysine was (Life Technologies) was supplemented with their isotopically labeled counterparts. The isotopic label was “heavy” when the medium was supplemented with 4, 4, 5, 5-D4 l-Lysine-2HCL (0.4 mM) and ^13^C_6_N_4_ l-Arginine (0.8 mM), “medium” when supplemented with ^13^C_6_^15^N_2_ l-Lysine-2HCL (0.4 mM) and ^13^C_6_ l-Arginine-HCL (0.8 mM) and “light” when supplemented with l-Lysine-2HCL (0.4 mM) and l-Arginine-HCL (0.8 mM). The resulting “heavy”, “medium”, and “light” SILAC media were supplemented with 10% SILAC FBS.

About 3 × 10^5^ fibroblasts were cultured for at least six divisions (8–15 days depending on their proliferation rate) in their corresponding media. CAFs were cultured in “Heavy” while NAFs in “Medium” medium. In the same time, co-cultures of 3 × 10^5^ CAFs with 1.2 × 10^5^ HCT116 and 3 × 10^5^ NAFs with 1.2 × 10^5^ HCT116 were cultured with “Light” medium.

Cells were washed five times with PBS and then cultured for 48 h with serum-free “Light” medium in order to eliminate all traces of serum in the cultures. After 48 h, the “Light” conditioned medium (CM) of the co-cultures was collected. “Heavy” CAFs and “Medium” NAFs were also washed and starved in 7 ml serum-free “Heavy” and “Medium” media, respectively; and incubated at 37 °C, 5% CO_2_. After 48 h, 3 ml of the “Light” CM (co-culture CAFs-HCT116) was added to the “heavy” CAFs on 10 cm 30 kPa plates and 3 ml of the CM from the co-culture NAFs-HCT116 was added to the “medium” NAFs, respectively. Using this procedure, we aimed to stimulate CAFs and NAFs with CM coming from co-cultures of cancer cells and fibroblasts, therefore containing all the cross talk-derived molecules.

After 48 h, the CM of CAFs and NAFs was collected and mixed in equal numbers accordingly with the number of cells, in order to have a 1:1 ratio. Mixed CM was filtered through 0.20 µm pore size filters and analyzed by mass spectrometry. For the total proteome analysis, “Heavy” CAFs and “Medium” NAFs were detached from their substrate using trypsin, mixed in equal numbers, spun down (1000 rpm for 3 min), washed with cold PBS, and lysed with lysis buffer for 10 min at 4 °C. The lysates were pelleted for 10 min at 4 °C and the supernatant was analyzed by mass spectrometry. The stable isotope labeling ratio was calculated by using a fraction of the different “cells” and analyzing by LC-MS/MS after protein in-gel separation and digestion of a blue band. The incorporation rates calculated from all quantified proteins were above 95%.

Secretomes (CM) of CAFs and NAFs were mixed at a 1:1 ratio according to cell number in cultures from which the secretome was prepared (total volume = 10 mL) and concentrated to 500 µL on Amicon Ultra-15, 10,000 molecular weight cutoff centrifugation filter units. To 500 µL of concentrated secretomes, 60 mg of urea and 16 µL of 1 M dithiotreitol were added, mixed on Nanosep (10 KDa, Pall) devices, and incubated at 57 °C for 15 min. The mixture was spun down and washed twice with 200 µL of 2 M urea in 0.1 Tris/HCl pH 8.5. About 100 µL of 0.05 M iodoacetamide was added and left for 30 min at RT in the dark. Two washes with 25 mM ammonium bicarbonate were performed and finally 5 µg trypsin/LysC (Promega) was added and subjected to 4 h digestion at 37 °C. The digested peptides were collected by centrifugation, and the filtrate was dried in a vacuum concentrator at room temperature and re-dissolved in solvent A (2% acetonitrile, 0.1% formic acid). Peptides were then subjected to liquid chromatography (LC)/mass spectrometry (MS) analysis. For proteome analysis, total proteins lysates of CAFs and NAFs were mixed at a 1:1 protein ratio and were separated by SDS–PAGE, and were digested in-gel with trypsin/LysC (Promega) as described in standard protocols. Extracted peptide was dried in a vacuum concentrator at room temperature and re-dissolved in solvent A before LC/MS analysis.

For the CAFs from patient 1, peptides were separated by reverse-phase chromatography using a nanoflow liquid chromatography (LC) system (Ultimate 3000, Dionex) with a 180-min two-step linear gradient of water/acetonitrile. For secretome sample analysis, peptides were eluted with a 900-min three step linear gradient. LC was coupled online to an LTQ-Orbitrap XL mass spectrometer (Thermo Fisher Scientific). Fragmentation of the top five peptides in each scan was done by collision-induced dissociation and the resulting fragments were analyzed in the linear trap (LTQ). Exclusion duration of 180 s was used and lock-mass option was enabled. For the CAFs from patient 2, peptides were analyzed by nanoLC-MS/MS using an RSLCnano system (Ultimate 3000, Thermo Scientific) coupled to an Orbitrap Fusion mass spectrometer (Q-OT-qIT, Thermo Fisher Scientific). Samples were loaded on a C18 precolumn (300 µm inner diameter × 5 mm; Dionex) at 20 µL/min in 2% acetonitrile, 0.05% TFA. After a desalting for 3 min, the precolumn was switched on the C18 column (75 μm i.d. × 50 cm, packed with C18 PepMap, 3 μm, 100 Å; LC Packings) equilibrated in solvent A. Bound peptides were eluted using a 280 min (from 1 to 40% (v/v)) linear gradient of solvent B (100% acetonitrile, 0.085% formic acid) for secretomes and 100 min (from 1 to 40% (v/v)) linear gradient of solvent B for proteomes, at a 400 nL/min flow rate and an oven temperature of 40 °C. We acquired Survey MS scans in the Orbitrap on the 400–1500 *m/z* range with the resolution set to a value of 120,000 and a 4 × 105 ion count target. Each scan was recalibrated in real time by co-injecting an internal standard from ambient air into the C-trap. Tandem MS was performed by isolation at 1.6 Th with the quadrupole, HCD fragmentation with normalized collision energy of 35, and rapid scan MS analysis in the ion trap. The MS2 ion count target was set to 104 and the max injection time was 100 ms. Only those precursors with charge state 2–7 were sampled for MS2. The dynamic exclusion duration was set to 60 s with a 10 ppm tolerance around the selected precursor and its isotopes. The instrument was run in top speed mode with 3 s cycles.

Data analysis was performed at the Institut Curie laboratory of mass spectrometry proteomics. Raw MS files from the Orbitrap were analyzed via Sequest HT with Proteome Discoverer (1.4, Thermo Scientific) using the Uniprot Human database (032015). Enzyme specificity was set to trypsin and a maximum of two miss cleavages was allowed. Oxidized methionine, N-terminal acetylation, carbamidomethyl cysteine, label 13C615N2 Lysine, and label 2H4 lysine were set as variable modifications. For identification, the false discovery rate (FDR) was set to 1% with Percolator *q*-values. The resulting files were further processed by using the Institut Curie-developed software myProMS^38^ version 3.0 (work in progress), which performs search engine results validation, false positive rate (FDR)-based data filtering, protein quantification, statistical analysis, and data visualization. For SILAC-based protein quantification, peptides XICs (extracted ion chromatograms) were retrieved from Proteome Discoverer. Scale normalization was applied to compensate for mixing errors of the different SILAC cultures as described by Yang et al.^39^ Protein ratios were computed as the geometrical mean of related peptides. To estimate ratio significance, a *t*-test was performed with a Benjamin–Hochberg FDR control threshold set to 0.05.

### Gene array analysis

About 2 × 10^5^ HCT116 cancer cells were cultured atop of 8 µm pore filters of a six-well plate (Corning) containing 10^5^ CAFs plated in the bottom chamber of the transwell in DMEM containing 10% FBS. After 12 h, the medium was changed to serum-free DMEM. For controls, CAFs were not present in bottom chambers. After 7 days, cancer cells were collected and washed with PBS.

Total RNA were purified using miRNeasy (Qiagen) using the manufacturer’s protocol. Extracted RNA was quantified using a spectrophotometer (Nanodrop ND1000, Thermo, Courtaboeuf, France) and RNA integrity was assessed by capillary electrophoresis (Bioanalyzer, RNA 6000 Nano total RNA Kit, Les Ulis, France). About 100 ng of total RNA was amplified, converted to complimentary DNA (cDNA), and labeled according to Affymetrix recommendations based on the WT. Amplified molecules were controlled after purification steps to monitor yields and sized of molecules. The labeled cDNA was hybridized and analyzed on Affymetrix GeneChip Human Gene 2.1 ST arrays, using the Genetitan device at the Genomic platform of Institut Curie. The data were analyzed by Genosplice (Paris).

### Statistical analysis

All experiments were performed in triplicates in 1–3 independent experiments. All statistical analysis and graphic representations were performed using SigmaPlot/SigmaStat or GraphPad Prism software. Data are represented as box and whiskers (10–90 percentile) plus outliers. Statistical significance was determined with one-way analysis of variance (ANOVA), Kruskal–Wallis test was applied; pairwise comparison (Dunn’s, Holm–Sidak, or Tuckey Method) as indicated; ***p < 0.0001; **p < 0.001; *p < 0.05.

### Data availability

The mass spectrometry proteomics data have been deposited to the ProteomeXchange Consortium via the PRIDE partner repository with the data set identifier PXD003670. The gene array data have been deposited in NCBI’s Gene Expression Omnibus and are accessible through GEO Series accession number GSE78947 (http://www.ncbi.nlm.nih.gov/geo/query/acc.cgi?acc=GSE78947). All analyzed data are available within the article and Supplementary Files, or available from the authors upon request.

## Electronic supplementary material

Supplementary Information

Peer Review File

Description of Additional Supplementary Files

Supplementary Movie 1

Supplementary Movie 2

Supplementary Movie 3

Supplementary Movie 4

Supplementary Movie 5

Supplementary Movie 6

Supplementary Movie 7

Supplementary Movie 8

Supplementary Data 1

Supplementary Data 2

Supplementary Data 3

Supplementary Data 4

Supplementary Data 5

Supplementary Data 6

Supplementary Data 7

Supplementary Data 8

Supplementary Data 9

Supplementary Data 10

Supplementary Data 11

Supplementary Data 12
