# Author Correction: Cancer-associated fibroblasts induce metalloprotease-independent cancer cell invasion of the basement membrane

**DOI:** 10.1038/s41467-018-03304-x

**Published:** 2018-03-07

**Authors:** Alexandros Glentis, Philipp Oertle, Pascale Mariani, Aleksandra Chikina, Fatima El Marjou, Youmna Attieh, Francois Zaccarini, Marick Lae, Damarys Loew, Florent Dingli, Philemon Sirven, Marie Schoumacher, Basile G. Gurchenkov, Marija Plodinec, Danijela Matic Vignjevic

**Affiliations:** 1 Institut Curie, PSL Research University, CNRS, UMR 144, F-75005 Paris, France; 2 Biozentrum, University of Basel, CH-4056 Basel, Switzerland; 3 Institut Curie, Department of surgical oncology, Curie Institute, F-75005 Paris, France; 4 Institut Curie, PSL Research University, Laboratoire de spectrométrie de masse protéomique, F-75005 Paris, France; 5 Institut Curie, PSL Research University, CNRS, U830, F-75005 Paris, France; 6 Institute of Pathology, University Hospital Basel, CH-4031 Basel, Switzerland

Correction to: *Nature Communications* 10.1038/s41467-017-00985-8, published online 13 October 2017

In the original version of this Article, financial support and contributions in manuscript preparation were not fully acknowledged. The PDF and HTML versions of the Article have now been corrected to include the following:

‘M.P. and P.O. would like to thank Prof. Roderick Y.H. Lim for advice during manuscript preparation and for providing the laboratory and microscopy infrastructure.… [We also thank] the NanoteraProject, awarded to the PATLiSciII Consortium (M.P and P.O)…’

