# Supplementary material for: Cancer-associated fibroblasts induce metalloprotease-independent cancer cell invasion of the basement membrane
Source: Nat Commun. 2017 Oct 13;8:924. doi: 10.1038/s41467-017-00985-8 (PMC5640679; doi:10.1038/s41467-017-00985-8)
Supplement: Supplementary file 1 — Supplementary Information [file 41467_2017_985_MOESM1_ESM.pdf]

Supplementary Information for **Carcinoma-associated fibroblasts induce metalloprotease-independent cancer cell invasion of the basement membrane** by Glentis et al.

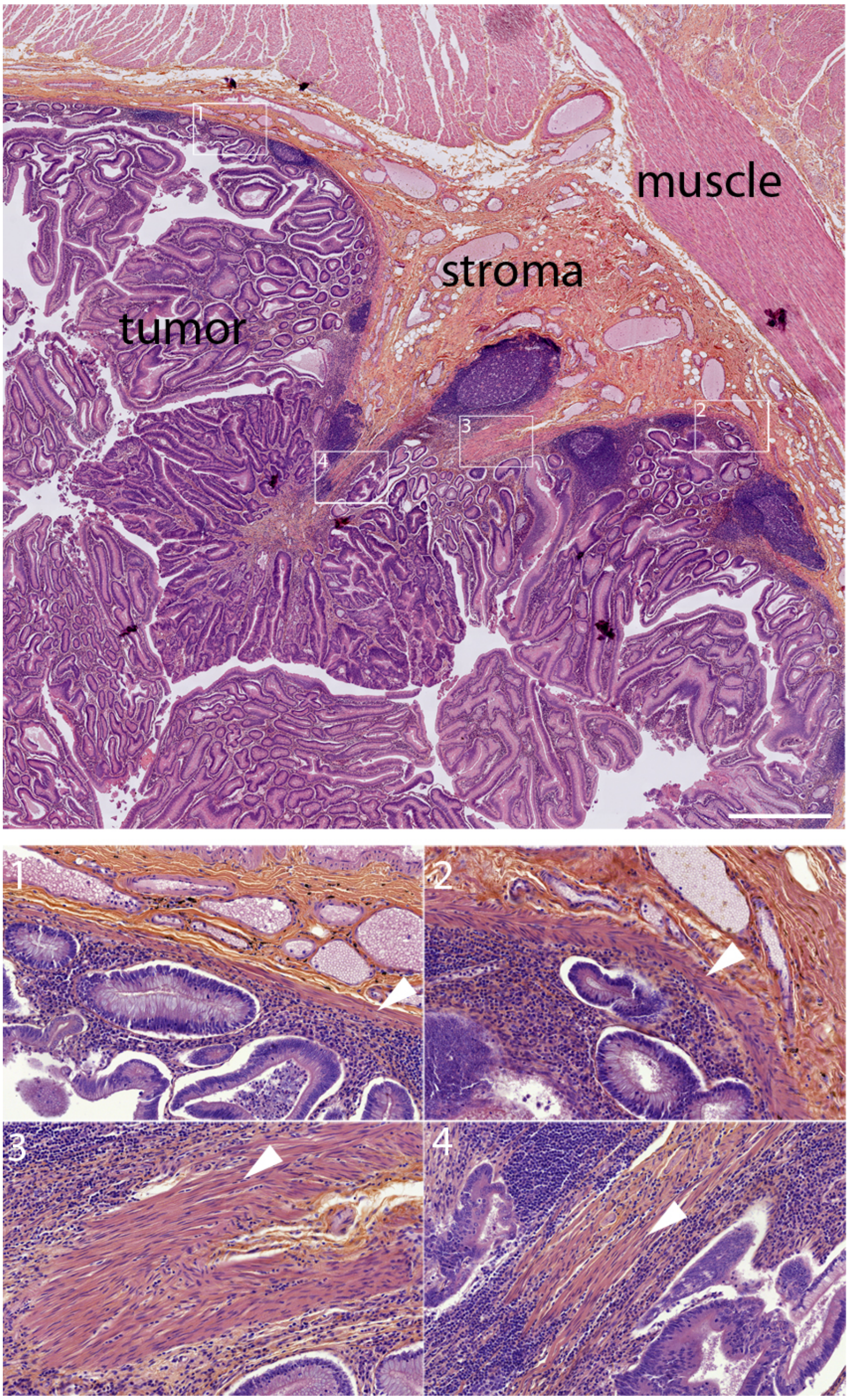

Supplementary Figure 1. Human colon carcinoma in situ

Hematoxylin/eosin staining of adjacent serial section of human carcinoma presented on Figure 1a. Scale bar, 1000µm. Insets, higher magnification of boxed regions. Arrows indicate αSMA positive cells.

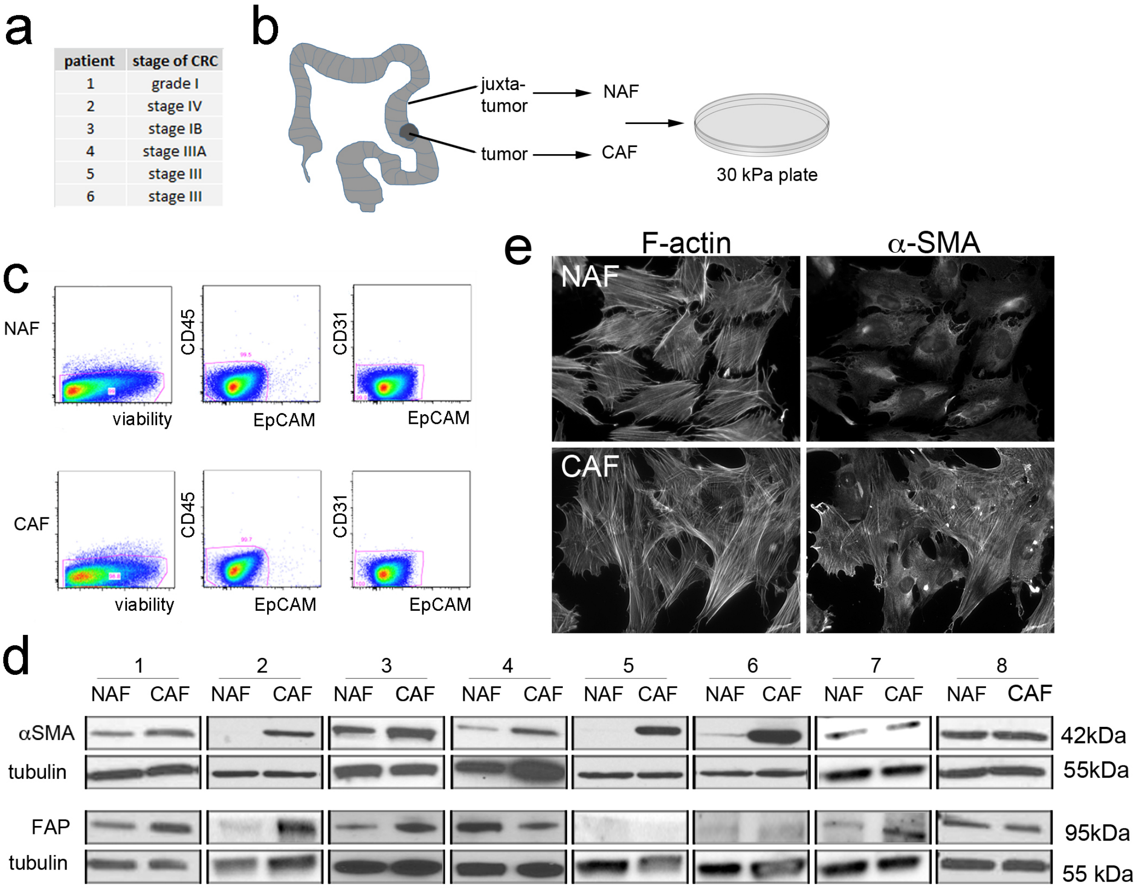

patient	stage of CRC
1	grade I
2	stage IV
3	stage IB
4	stage IIIA
5	stage III
6	stage III

Supplementary Figure 2. Isolation and characterization of human primary colon fibroblasts

a. Stage of human colon carcinoma used for CAFs isolation

b. Tumors and juxta-tumoral tissues were mechanically dissociated into 1mm pieces. After 2-3 weeks fibroblasts emerged from tissue pieces. Cells were then plated on collagen-coated plates with 30kPa stiffness.

c. FACS analysis showing that isolated cells were negative for epithelial, immune cells and endothelial markers, such as EpCAM, CD45 and CD31, respectively. Example from patient 1.

d. Characterization of the fibroblasts isolated from eight patients by Western blot. Vimentin was used as a marker of fibroblasts, whereas αSMA and FAP were used as CAF markers.

e. F-actin stained with phalloidin and αSMA with specific antibodies in CAF and NAF.

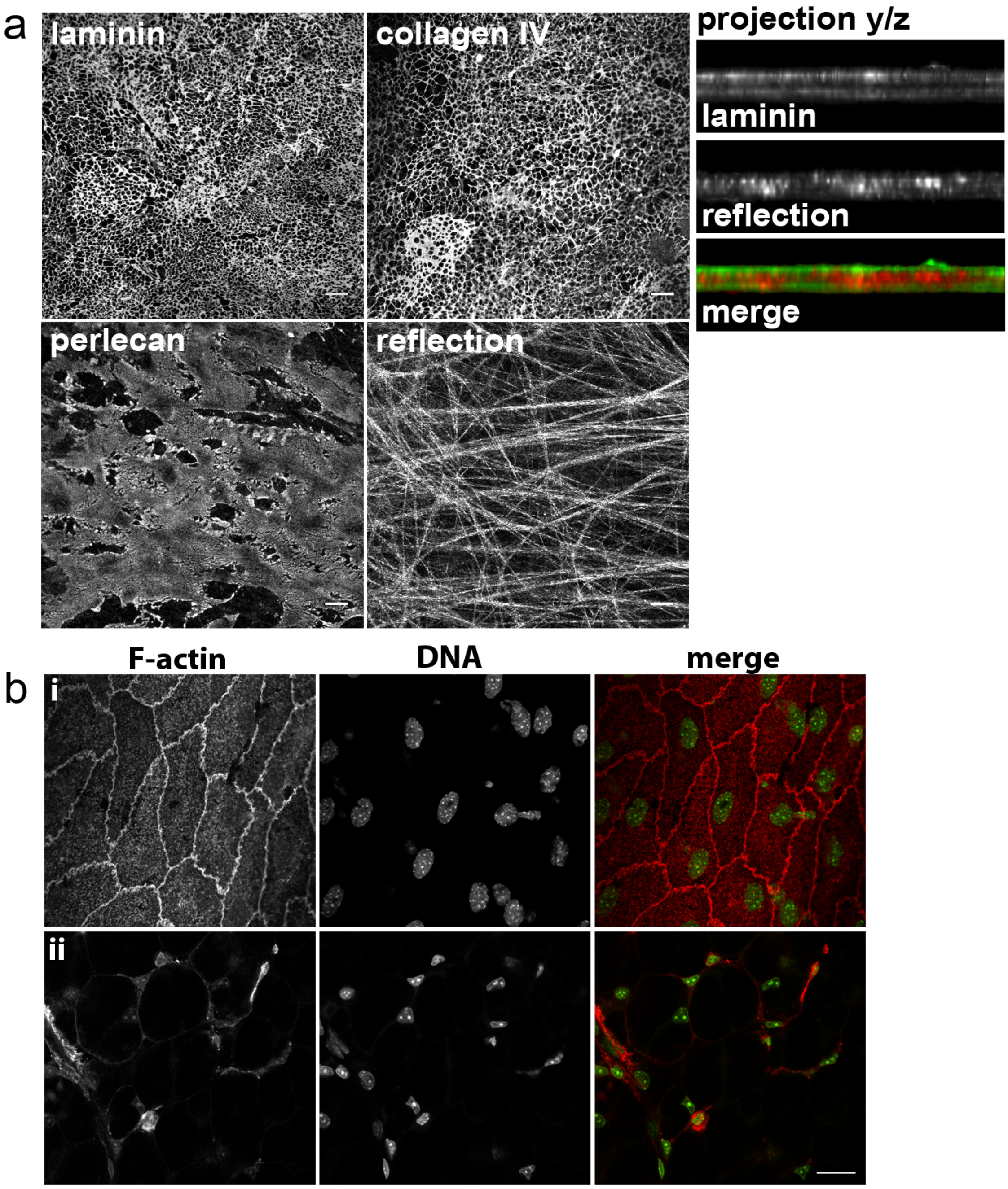

Supplementary Figure 3. Cellular and molecular composition of a mouse mesentery

a. Extracellular components of the mouse mesentery BM (laminin, type IV collagen and perlecan) revealed by immunofluorescence. The fibrillar network of ECM proteins present in between 2 laminin layers is revealed by reflection microscopy. Scale bar, 20µm.

b. At least two types of resident mesothelial cells are present in a mouse mesentery: a monolayer of cells with big nuclei that covers the mesentery (i) and cells with small nuclei and thin protrusions embedded in a fibrillar ECM network (ii). Actin cytoskeleton (phalloidin, red). DNA (DAPI, green). Scale bar, 20µm.

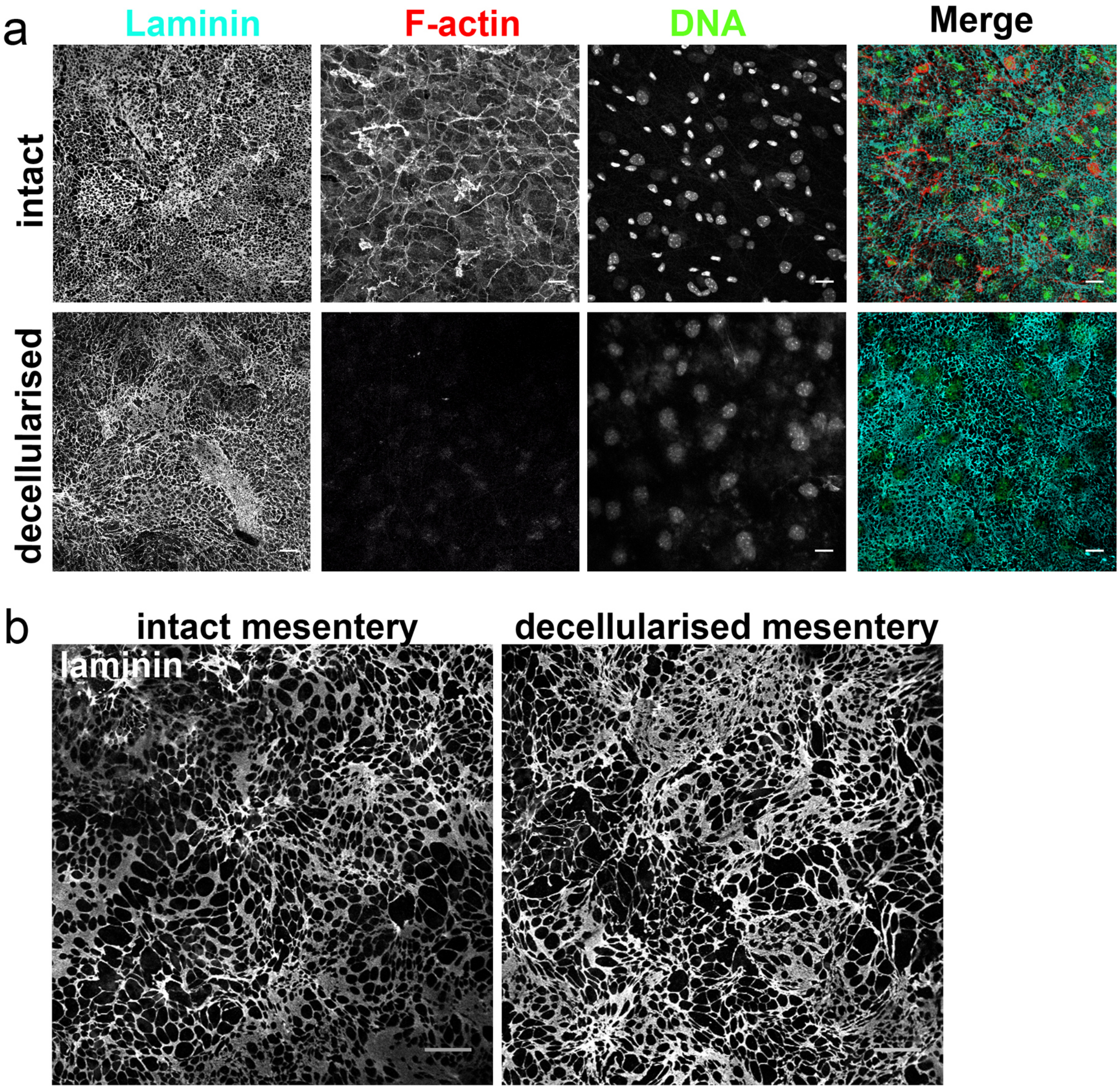

Supplementary Figure 4. Comparison of intact and decellularized mouse mesenteries

a. The mesentery was decellularized using ammonium hydroxide. Cell debris was visualized by staining the actin cytoskeleton (phalloidin, red) and the DNA (DAPI, green). Ammonium hydroxide treatment did not affect the organization of the ECM as revealed by laminin staining (cyan).

b. Higher magnification of the laminin network in the ammonium hydroxide-treated and non-treated mesentery. Scale bar, 20µm.

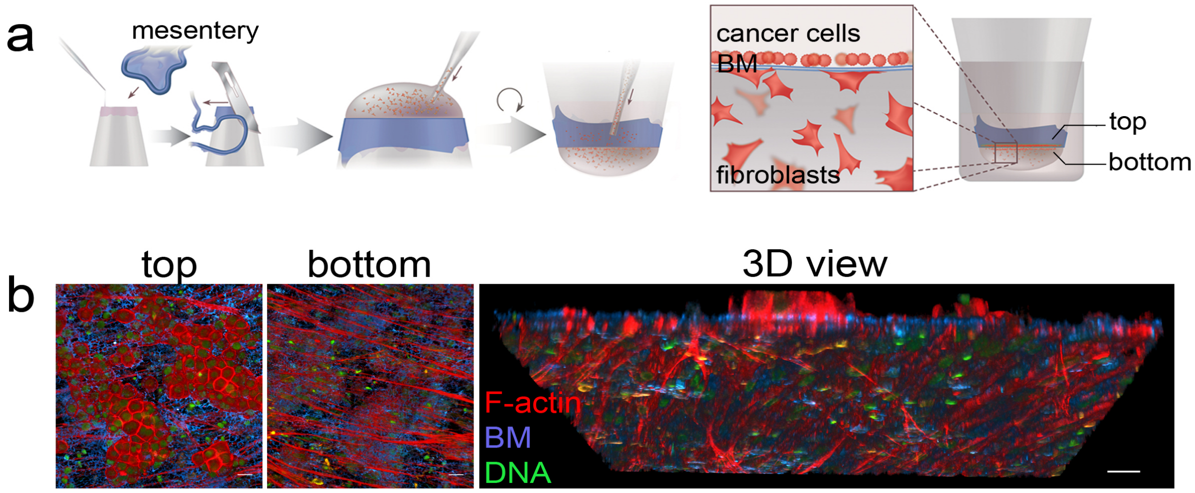

Supplementary Figure 5. Co-culture of colon cancer cells HT29 and CAF on mouse mesentery

a. Schematic representation of the preparation of the co-culture assay using the native basement membrane. Mesothelium was isolated from mouse (intestine shown in blue) and glued to the plastic holder. On the bottom side of the mesentery a drop of collagen type 1 containing fibroblasts was added. Once polymerized, cancer cells were seeded on the top of the mesentery. Cells were co-cultured for 10-25 days in the media containing 1% serum, thus cell growth and invasion were mostly a result of the crosstalk between cells.

b. Top, bottom and 3D view of human colon cancer cells HT29 were cultured atop of mouse mesentery (top) for 25 days in the presence of CAF from patient 1 embedded in type I collagen matrix on the other side of the mesentery (bottom). Cells visualized by staining the actin cytoskeleton (phalloidin, red) and DNA (DAPI, green). The mesentery is detected by laminin staining (cyan). Right panel, x/y/z maximal projection. Scale bars, 20 µm.

Proteins enriched in **CAFs**

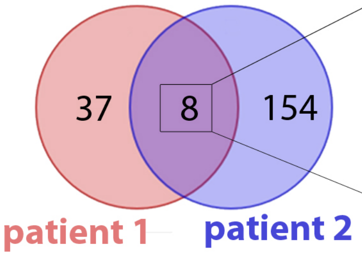

	CAF 1		CAF 2	
Acronyms	fold change	p-value	fold change	p-value
KPYM *	1,67	0,0004801	1,55	2,153E-05
FINC	1,74	1,414E-06	1,8	4,307E-13
AMPN	2,36	0,0001365	1,56	1,236E-29
SERPH *	2,2	0,0496335	2	0,0100967
CO6A1	2,22	3,137E-08	2,56	6,348E-17
LAMB2	1,52	0,0385078	3,61	1,317E-07
CO6A2	2,32	0,003558	2,62	1,829E-10
ADDA	1,51	0,0418749	1,75	5,363E-07

Proteins decreased in **CAFs**

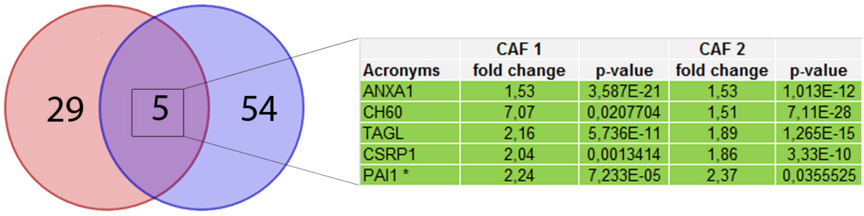

	CAF 1		CAF 2	
Acronyms	fold change	p-value	fold change	p-value
ANXA1	1,53	3,587E-21	1,53	1,013E-12
CH60	7,07	0,0207704	1,51	7,11E-28
TAGL	2,16	5,736E-11	1,89	1,265E-15
CSRP1	2,04	0,0013414	1,86	3,33E-10
PAI1 *	2,24	7,233E-05	2,37	0,0355525

Supplementary Figure 6. Proteomic comparison of CAFs and NAFs by SILAC

Comparative analysis of proteins present in higher or lower amounts in total proteomes (including secretome) of CAFs compared to their paired NAFs from 2 patients (patient 1 and 2). Venn diagram of differentially enriched or reduced proteins in CAFs compared to their NAFs in the two different patients. Numbers in the intersection of the Venn diagram represent the proteins that are present in higher (pink) or lower (green) amounts in CAFs in both patients compared to their paired NAFs. For each protein fold change and p-value is presented. Peptide ratios with a p-value ≤ 0.05 are reported as significant.

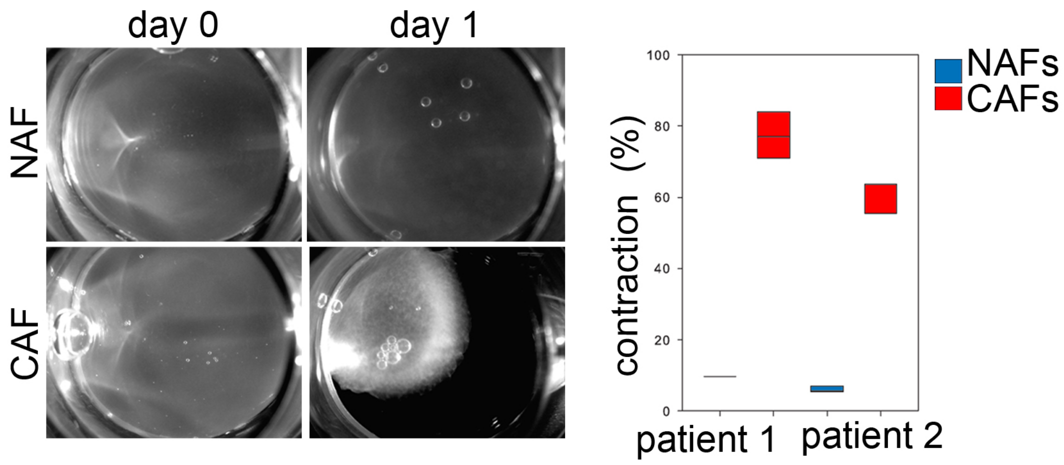

Supplementary Figure 7. Fibroblasts contraction assay

Left: Contraction of collagen plugs by NAF and CAF from patient 2. Day 0, fibroblasts were embedded in collagen gels, Day 1 collagen plugs after one day of culture.

Right: Quantification of collagen contraction, calculated as a decrease in size of the collagen gel in % at day 1 from the initial size at day 0. n=1-3 collagen plugs for each condition from 1 experiment. *, p<0,05; ANOVA, Kruskal-Wallis method.

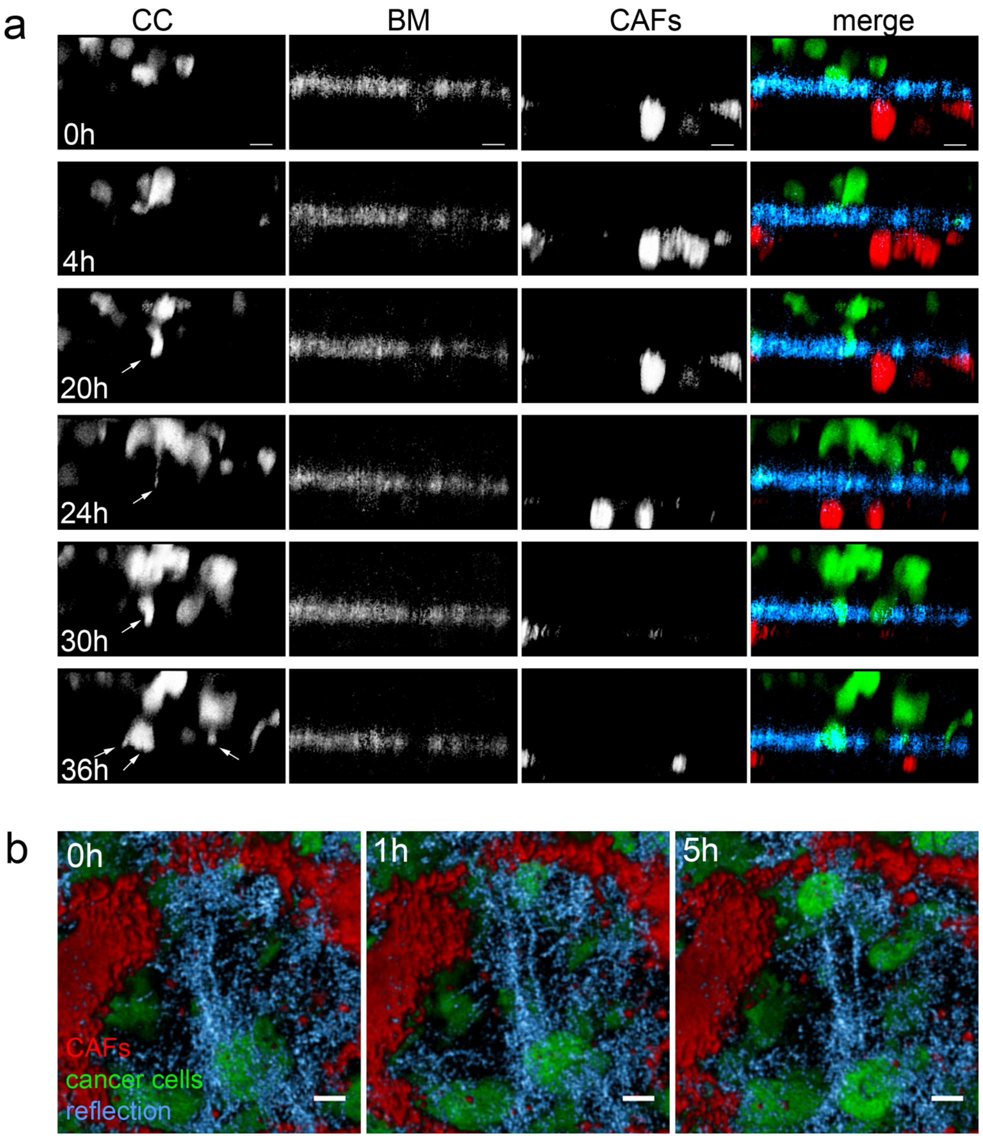

Supplementary Figure 8. Time-lapse analysis of BM invasion at early time points

a. y/z resliced images of co-cultures of cancer cells expressing cytoplasmic GFP (green) and CAFs from patient 1 labeled with vital dye (red) over time (in hours). Mesentery revealed by reflection (cyan). Scale bars, 20µm. Arrows indicate movement of fibroblasts. Dashed line – eye guide representing BM.

b. x/y montage of co-cultures of cancer cells expressing cytoplasmic GFP (green) and CAFs from patient 1 labeled with vital dye (red) over time (in hours). Mesentery revealed by reflection (cyan). Scale bars, 20µm. Imaging started after 2 days of co-culture. Arrow indicate cancer cell protrusions that penetrate the BM.
