# Supplementary material for: Cancer-associated fibroblasts induce metalloprotease-independent cancer cell invasion of the basement membrane
Source: Nat Commun. 2017 Oct 13;8:924. doi: 10.1038/s41467-017-00985-8 (PMC5640679; doi:10.1038/s41467-017-00985-8)
Supplement: Supplementary file 3 — Description of Additional Supplementary Files [file 41467_2017_985_MOESM3_ESM.pdf]

Description of Additional Supplementary Files

File Name: Supplementary Movie 1
Description: Co-culture of HT29 cancer cells and CAFs on the BM 3D representation of human colon cancer cells HT29 cultured atop of mouse mesentery for 25 days in the presence of CAF from patient 1 embedded in type I collagen matrix on the other side of the mesentery. Cells visualized by staining the actin cytoskeleton (phalloidin, red) and DNA (DAPI, green). The mesentery is detected by laminin staining (cyan).

File Name: Supplementary Movie 2
Description: Co-culture of HCT116 cancer cells and CAFs on the BM 3D representation of human colon cancer cells HCT116 cultured atop of mouse mesentery BM for 10 days in the presence of CAFs from patient 1 embedded in type I collagen matrix on the other side of the mesentery. Cells visualized by staining the actin cytoskeleton (phalloidin, red) and DNA (DAPI, green). The mesentery is detected by reflection (blue).

File Name: Supplementary Movie 3
Description: 3D view of BM invasion over time Time-lapse imaging of HCT116 and CAFs co-culture on the BM. Cancer cells express cytoplasmic GFP (green) and CAFs from patient 1 are labeled by vital dye (red). Mesentery revealed by reflection (cyan). Imaging started after 5 days of co-culture. Time, hours.

File Name: Supplementary Movie 4
Description: Early time points of cancer cells invasion in presence of CAFs, x/y view Time-lapse imaging of HCT116 and CAFs on the BM. Cancer cells expressing cytoplasmic GFP (green) and CAFs from patient 1 labeled with vital dye (red). Mesentery revealed by reflection (grey). Imaging started after 2 days of co-culture and lasted for 67h. Time, hours. x/y view.

File Name: Supplementary Movie 5
Description: Early time points of cancer cells invasion in presence of CAFs, y/z view Time-lapse imaging of HCT116 and CAFs on the BM. Cancer cells expressing cytoplasmic GFP (green) and CAFs from patient 1 labeled with vital dye (red). Mesentery revealed by reflection (blue). Imaging started after 2 days of co-culture and lasted for 67h. Time, hours. y/z view.

File Name: Supplementary Movie 6
Description: Late time points of cancer cells invasion in presence of CAFs, x/y view Time-lapse imaging of HCT116 and CAFs on the BM. Cancer cells expressing cytoplasmic GFP (green) and CAFs from patient 1 labeled with vital dye (red). Mesentery revealed by reflection (grey). Imaging started after 5 days of co-culture and the total duration of the movie was 45h. Time, hours. x/y view.

File Name: Supplementary Movie 7
Description: Late time points of cancer cells invasion in presence of CAFs, y/z view Time-lapse imaging of HCT116 and CAFs on the BM. Cancer cells expressing cytoplasmic GFP (green) and CAFs from patient 1 labeled with vital dye (red). Mesentery revealed by reflection (blue). Imaging started after 5 days of co-culture and the total duration of the movie was 45h. Time, hours. y/z view.

File Name: Supplementary Movie 8
Description: CAFs stretching the BM Time-lapse imaging of HCT116 and CAFs on the BM after laser ablation. Cancer cells expressing cytoplasmic GFP (green) and CAFs from patient 1 labeled with vital dye (red). Mesentery revealed by reflection (blue). 100µm2 hole was created using two-photon

ablation after 5 days of co-culture and the total duration of the movie was 20h. Time, hours. Maximal projection.

File Name: Supplementary Data 1
Description: Differentially regulated genes in CAFs stimulated HCT116 cells. List of the 99 Regulated Genes between HCT116 cultured with CAFs vs. “naïve” HCT116 (Fold-change ≥ 1,5; P-Value ≤ 0,05). Algorithm version: EASANA 2014_1

File Name: Supplementary Data 2
Description: . Hierarchical Clustering of Regulated Genes a. Hierarchical Clustering by Gene Intensities. b. Hierarchical Clustering by Distance to Mean

File Name: Supplementary Data 3
Description: Pathway Analysis on Regulated Genes

File Name: Supplementary Data 4
Description: Transcription Factor Analysis

File Name: Supplementary Data 5
Description: Proteins upregulated in CAF1 proteome

File Name: Supplementary Data 6
Description: Proteins upregulated in CAF1 secretome

File Name: Supplementary Data 7
Description: Proteins downregulated in CAF1 proteome

File Name: Supplementary Data 8
Description: Proteins downregulated in CAF1 secretome

File Name: Supplementary Data 9
Description: Proteins upregulated in CAFs2 proteome

File Name: Supplementary Data 10
Description: Proteins upregulated in CAFs2 secretome

File Name: Supplementary Data 11
Description: Proteins downregulated in CAFs2 proteome

File Name: Supplementary Data 12
Description: Proteins downregulated in CAFs2 secretome
