# Supplementary material for: Cancer-associated fibroblasts induce metalloprotease-independent cancer cell invasion of the basement membrane
Source: Nat Commun. 2017 Oct 13;8:924. doi: 10.1038/s41467-017-00985-8 (PMC5640679; doi:10.1038/s41467-017-00985-8)
Supplement: Supplementary file 13 — Supplementary Data 2 [file 41467_2017_985_MOESM13_ESM.pdf]

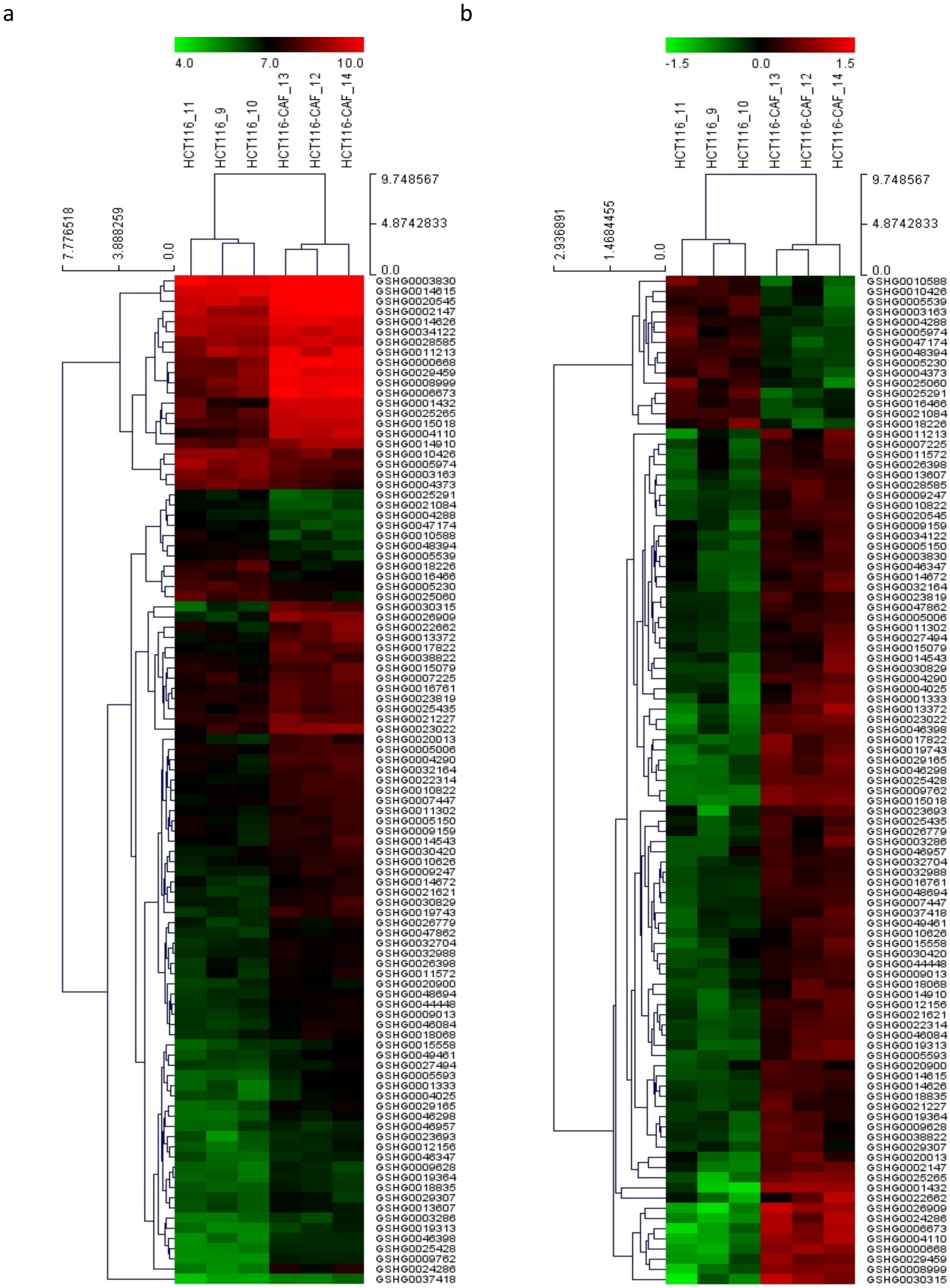

Supplementary Data 2. Hierarchical Clustering of Regulated Genes
a: Hierarchical Clustering by Gene Intensities. b: Hierarchical Clustering by Distance to Mean
