# Supplementary material for: Cancer-associated fibroblasts induce metalloprotease-independent cancer cell invasion of the basement membrane
Source: Nat Commun. 2017 Oct 13;8:924. doi: 10.1038/s41467-017-00985-8 (PMC5640679; doi:10.1038/s41467-017-00985-8)
Supplement: Supplementary file 2 — Peer Review File [file 41467_2017_985_MOESM2_ESM.pdf]

Editorial Note: this manuscript has been previously reviewed at another journal that is not operating a transparent peer review scheme. This document only contains reviewer comments and rebuttal letters for versions considered at Nature Communications.

REVIEWERS' COMMENTS:

Reviewer #1 (Remarks to the Author):

This work is significantly improved from the previous submission. It is now a compelling and interesting study, and I have no hesitation in recommending prompt publication.

Reviewer #2 (Remarks to the Author):

In the revised manuscript by Glentis et al. the authors have addressed all my comments.
The manuscript is improved from its original version. In particular, the authors have now included CAFs from additional patients, and their claims are more accurate (as reflected by the revised title).

Therefore, I now recommend publication of this interesting study.
